# Taxonomic treatment of three *Garcinia* species (section *Brindonia*, Clusiaceae) in Thailand, with six new synonyms and ten lectotypifications

**DOI:** 10.3897/phytokeys.270.172287

**Published:** 2026-01-28

**Authors:** Chatchai Ngernsaengsaruay, Pichet Chanton

**Affiliations:** 1 Department of Botany, Faculty of Science, Kasetsart University, Chatuchak, Bangkok 10900, Thailand Biodiversity Center, Kasetsart University (BDCKU) Bangkok Thailand; 2 Biodiversity Center, Kasetsart University (BDCKU), Chatuchak, Bangkok 10900, Thailand Faculty of Science, Kasetsart University Bangkok Thailand; 3 Suan Luang Rama IX Foundation, Nong Bon Subdistrict, Prawet District, Bangkok, 10250, Thailand Suan Luang Rama IX Foundation Bangkok Thailand

**Keywords:** Dioecy, edible plants, *Garcinia cowa* var. *cowa*, *Garcinia oliveri*, *Garcinia schomburgkiana*, Guttiferae, Malpighiales, synonymizations, taxonomy

## Abstract

The taxonomy of three *Garcinia* species, *G. cowa* var. *cowa*, *G. oliveri*, and *G. schomburgkiana* (section *Brindonia*, Clusiaceae), is revised for Thailand. All three species have edible fruits, young shoots, and leaves with a sour taste. Morphological descriptions and illustrations are provided, along with notes on distribution, phenology, conservation status, etymology, vernacular names, uses, specimens examined, habitats, and ecology. Four taxa, *G. cochinchinensis*, *G. fusca*, *G. nigrolineata*, and *G. plena*, are newly synonymized under *G. cowa* var. *cowa*, and two taxa, *G. delpyana* and *G. bancana* var. curtisii, are newly synonymized under *G. oliveri*. Ten names are lectotypified here, including six synonyms of *G. cowa* var. *cowa* (*G. cochinchinensis*, *G. fusca*, *G. kunstleri*, *G. loureiroi*, *G. nigrolineata*, and *G. plena*), *G. oliveri* and its two synonyms (*G. delpyana* and *G. curtisii*), and *G. schomburgkiana*. All three species have a conservation status of Least Concern (LC).

## Introduction

*Garcinia* L. is a group of evergreen trees, occasionally shrubs, that are usually dioecious but sometimes polygamo-dioecious (also known as trioecious). It also includes some obligately and facultatively agamospermous species (Ngernsaengsaruay et al. 2023a, 2023b). The color of exudate secreted from cut boles, twigs, leaves, and fruits can be yellow, pale yellow, white, cream, or clear (Ngernsaengsaruay et al. 2022a). The genus comprises at least 250 species (Stevens 2007) and possibly as many as c. 400 species (Gaudeul et al. 2024; POWO 2025), making it the largest genus in Clusiaceae Lindl. (Guttiferae Juss.) and Malpighiales Juss. ex Bercht. & J. Presl. It is a pantropically distributed genus, with centers of diversity located in Africa (Madagascar), Australasia, and Southeast Asia (Sweeney and Rogers 2008; Gaudeul et al. 2024). In Asia, *Garcinia* is most diverse in the Malesian Region but also extends north to southern China, west to India, and east to the Micronesian islands (Nazre et al. 2018).

*Garcinia* sect. *Brindonia* (Thouars) Choisy, recently updated by Gaudeul et al. (2024) with 91 recognized species, is the largest section of the genus. Species in this section are distributed in Madagascar, Indomalaya, tropical Australasia, and Oceania (Gaudeul et al. 2024). The section is characterized by flowers with four sepals and four petals; staminate flowers usually without a pistillode [except in species such as *G. atroviridis* Griff. ex T. Anderson, *G. pedunculata* Roxb. ex Buch.-Ham., and *G. sopsopia* (Buch.-Ham.) Mabb.]; stamens united into a single central bundle (or into a ring when a pistillode is present, as in *G. atroviridis*); 4-thecous anthers, rarely 2-thecous; multilocular ovaries; stigmas divided into as many distinct rays as there are ovary locules and usually papillate; fruits in many species with furrows or grooves along the septal radii; and terminal or axillary inflorescences with one to many flowers (Jones 1980; Ngernsaengsaruay 2022; Gaudeul et al. 2024; Ngernsaengsaruay et al. 2025a). The fruits or leaves of many species in this section are edible, including *G. atroviridis*, *G. lanceifolia* Roxb., *G. pedunculata*, *G. siripatanadilokii* Ngerns., Meeprom, Boonth., Chamch. & Sinbumr., and *G. sopsopia* (Buch.-Ham.) Mabb. (Ngernsaengsaruay 2022; Ngernsaengsaruay et al. 2022b; Ngernsaengsaruay et al. 2025a).

In Thailand, the genus *Garcinia* was enumerated by Craib (1925) with 20 species. Gardner recorded six species in northern Thailand (Gardner et al. 2000) and 23 species (including five unidentified species) in Peninsular Thailand (Gardner et al. 2015). A taxonomic revision of *Garcinia* in Thailand has recently been undertaken by the first author as part of the Flora of Thailand project, and treatments for several sections have been produced (Ngernsaengsaruay and Suddee 2016, 2022; Ngernsaengsaruay 2022; Ngernsaengsaruay et al. 2022a, 2022b, 2023a, 2023b, 2024a, 2024b; Ngernsaengsaruay and Chanton 2024; Ngernsaengsaruay et al. 2025a, 2025b).

In this paper, we provide a taxonomic treatment of three *Garcinia* species in sect. *Brindonia* in Thailand: *G. cowa* Roxb. ex Choisy var. *cowa*, *G. oliveri* Pierre, and *G. schomburgkiana* Pierre. This treatment includes synonymizations, lectotypifications, morphological descriptions, and illustrations, along with notes on distribution, phenology, preliminary conservation assessments, etymology, vernacular names, uses, specimens examined, habitats, and ecology.

## Materials and methods

Collected specimens were examined through consultation of the literature (e.g., Anderson 1874; Kurz 1874, 1877; Pierre 1882, 1883; Vesque 1889, 1893; King 1890; Pitard 1910; Craib 1924; Gagnepain 1943; Maheshwari 1964; Kochummen and Whitmore 1973; Whitmore 1973; Jones 1980; Long 1984; Singh 1993; Li et al. 2007; Mohanan et al. 2023) and by comparison with herbarium specimens deposited in the following herbaria: AAU, BK, BKF, BM, C, CMUB, K, P, PSU, QBG, and SING, as well as those available in the virtual herbarium databases of A, AAU, BM, CAL, E, G, GH, K, L (including U), P, S, and TCD, the Wallich Catalogue Online, and via GBIF and JSTOR. All herbarium acronyms follow Thiers (2025, continuously updated). All specimens cited were seen by the authors unless stated otherwise. The taxonomic history of the species was compiled using both the literature and online databases (IPNI 2025; POWO 2025; WFO 2025). Morphological characteristics, distributions, habitats and ecology, phenology, and uses were described from historic and newly collected herbarium specimens, as well as from the authors’ observations during fieldwork. Vernacular names were compiled from the specimens examined and relevant literature (e.g., Pooma and Suddee 2014). Thailand’s floristic regions follow *Flora of Thailand* Vol. 4(3.3) (The Forest Herbarium, Department of National Parks, Wildlife and Plant Conservation 2023). Conservation status assessments were conducted following the IUCN Red List Categories and Criteria (IUCN Standards and Petitions Committee 2024), combined with GeoCAT analysis (Bachman et al. 2011) and field information.

## Results and discussion

### Taxonomic treatment

#### Garcinia cowa

Taxon classificationPlantaeMalpighialesClusiaceae

Roxb. [Hort. Bengal. 42. 1814] ex Choisy, Mém. Nouv. Gen. Guttif.: 17. 1823.

05D93747-6F21-5264-A729-46A426500B62

≡ Stalagmitis cowa (Roxb. ex Choisy) G. Don, Gen. Hist. 1: 621. 1831. Type. East Indies [India], s.d., *W. Roxburgh s.n*. (lectotype: first-step designated by Maheshwari (1964), BR [without barcode]; second-step designated here: BR digital image! [BR0000005108138]; isolectotypes: BR digital images! [BR0000006912420, BR0000006915667]).
= Garcinia cochinchinensis (Lour.) Choisy, Mém. Nouv. Gen. Guttif.: 17. 1823; Pierre, Fl. Forest. Cochinch. 1(5): 28. 1883.— Oxycarpus cochinchinensis Lour., Fl. Cochinch. 2: 648. 1790. Type. [Vietnam], Habitat: tam cultus, quam incultus in Cochinchinâ [southern Cochinchina]; noting that the species grows in both cultivated and uncultivated conditions, s.d., *J. de Loureiro s.n*. (lectotype: designated here, BM digital image! [BM000611621]; isolectotype: BM digital image! [BM000611622]), syn. nov.
= Oxycarpus gangetica Buch.-Ham., Mem. Wern. Nat. Hist. Soc. 5. 344. 1824.
= Garcinia lanceifolia Wall. [Numer. List: 171. Wallich Cat. 4861C. 1831, *nom. nud*.], non Roxb. 1832.
= Garcinia roxburghii Wight, Illustr. Ind. Bot. 1: 125. 1840, *pro parte*.
= Garcinia nigrolineata Planch. ex T. Anderson in Hook. f., Fl. Brit. India 1(2): 263. 1874; Pierre, Fl. Forest. Cochinch. 1(5): 29, t. 81F. 1883. Type. Peninsular Malaysia, Malacca, s.d., *W. Griffith 854* (Distributed at Kew, 1861–1862) (lectotype: designated here, K! [K000677662]; isolectotypes: CAL digital image! [CAL0000005801], GH digital image! [GH00067491], L digital image! [L2417126], P! [P04701603], W digital image! [W-0073380], syn. nov.
= Garcinia loureiroi Pierre, Fl. Forest. Cochinch. 1(5): 28, t. 66. 1883. Type. [Vietnam], Culta ad pagum Cây bé austro gallicae Cochinchinae [cultivated near the village of Cây bé in southern Cochinchina], 20 Feb 1869, *J. B. L. Pierre 420* (lectotype: designated here, P digital image! [P05061502]; isolectotypes: P digital images! [P05061498, P05061501, P05061503]).
= Garcinia fusca Pierre, Fl. Forest. Cochinch. 1(5): 28, t. 67. 1883. Type. [Vietnam], ad flumen Saigon gallicae austro Cochinchinae [the banks of the Saigon River], 18 Apr 1866, *J. B. L. Pierre 3622* (lectotype: designated here, K! [K000742484]; isolectotypes: BM! [BM001191365], L digital images! [L0700338, L0700339], S digital image! [S11-34587]), syn. nov.
= Garcinia kunstleri King, J. Asiat. Soc. Bengal, Pt. 2, Nat. Hist. 59(2): 165. 1890. Type. Peninsular Malaysia, Perak, Tapa, s.d., *L. Wray 328* (lectotype: designated here, CAL digital image! [CAL0000005872]; isolectotype: CAL digital image! [CAL0000005873]).
= Garcinia plena Craib, Bull. Misc. Inform. Kew 1924(3): 85. 1924; Craib, Fl. Siam. 1(1): 117. 1925. Type. Thailand, Nan, Ban Tiu, 4 Mar 1921, *A. F. G. Kerr 5007* (lectotype: designated here, K! [K000677703]; isolectotypes: BK! [BK257947], BM! [BM000611604]), syn. nov.

#### Garcinia cowa

Taxon classificationPlantaeMalpighialesClusiaceae

Roxb. ex Choisy var. cowa, Rheedea 33(3): 123. figs 5, 6. 2023.

3DADC1D9-CF90-59D5-B791-AF7EDCEA5EEB

Figs 1, 2, 3

##### Description.

***Habit*** evergreen, occasionally briefly deciduous trees, dioecious, 5–20(–25) m tall, 25–140 cm GBH; exudate yellow and sticky; branches decussate, horizontal, or nearly horizontal, sometimes drooping; branchlets green, 4-angular, glabrous. ***Bark*** brown, dark brown, or dark grayish brown, rough, shallowly fissured, or scaly; inner bark red, pinkish red, or creamy white. ***Terminal bud*** concealed between the bases of the uppermost pair of petioles. ***Leaves*** decussate; lamina variable in shape and size, narrowly elliptic, elliptic, lanceolate, lanceolate-ovate or ovate, 5–15 × 3–6 cm (occasionally larger in some populations, 16–20 × 4–7 cm), apex acute or acuminate, base cuneate, margin entire or repand, subcoriaceous, dark green above, paler below, glabrous and glossy on both surfaces, midrib slightly raised (proximal part) and flattened (distal part) above, raised below, secondary veins 6–10 pairs, curving towards the margin and connected in distinct loops and united into an intramarginal vein, flattened above, slightly raised below, with intersecondary veins, veinlets reticulate, visible above, interrupted long wavy lines (glandular wavy lines, also called exudate containing canals) present, of differing lengths, running across the secondary veins to the apex; petiole green, sometimes with the uppermost pair greenish red, turning green with age, 0.6–1.5 cm long, 2–5 mm diam., slightly raised above, glabrous, with a basal appendage clasping the branchlet; fresh leaves brittle when crushed; young leaves red or brownish red, turning pale green, glossy. ***Inflorescences*** terminal or borne on short, leafless lateral branchlets, in fascicles of 2–9-flowered cymes (staminate inflorescence usually bearing more flowers than pistillate ones), or sometimes a solitary flower (in pistillates); bracts early caducous. ***Flowers*** unisexual, 4-merous; pedicel greenish yellow to yellow, glabrous; bracteoles early caducous; sepals and petals decussate, pale yellow to yellow, glabrous; sepals concave; petals not concave, slightly thick and fleshy. ***Flower buds*** pale green, becoming greenish yellow to yellow before anthesis, subglobose or globose, 3–6 mm diam. ***Staminate flowers*** 0.8–1.2 cm diam.; pedicel 0.8–2 cm long, 2.5–3.5 mm diam.; sepals 4, broadly elliptic or elliptic, sometimes orbicular, 3.5–7 × 3–7 mm, the outer pair slightly larger than the inner pair, apex rounded; petals 4, broadly oblong, oblong, broadly elliptic or elliptic, 0.8–1.2 × 0.6–1 cm, subequal, apex rounded; stamens numerous, united into a single central 4-sided or weakly 4-lobed bundle surrounding a pistillode or without a pistillode, bundle 4–6.5 mm diam; filaments very short; anthers small, 4-thecous, longitudinally dehiscent; pistillode fungiform (mushroom-shaped), 1.5–2.5 mm long. ***Pistillate flowers*** 0.9–1.4 cm diam.; pedicel 3–5 mm long, 2–4 mm diam.; sepals and petals same as in staminate flowers; staminodes 18–27, united into 4 bundles, surrounding the ovary; pistil fungiform, 4.5–6.5 mm long; ovary pale green, subglobose or globose, 3.5–6 × 4–6.5 mm; stigma pale yellow, convex, radiate, shallowly 6–9-lobed, 3.5–5 mm diam., papillate. ***Fruits*** berries, green, turning yellow to orangish yellow when ripe, glabrous, cut fruits with a sticky yellow exudate, variable in shape, subglobose, globose, depressed globose, or broadly ellipsoid, 3–7 × 2.5–5 cm, sometimes oblique, asymmetrical, shallowly or weakly 6–9-lobed, without or with a short, thick beak at the apex, pericarp fleshy, 2–3.5 mm thick; persistent stigma dark brown, 3.5–6 mm diam., weakly 6–9-lobed, papillate; persistent sepals slightly larger than in flowering material; fruiting stalk short and thick, 3–6 mm long, 3–5.5 mm diam. ***Seeds*** 6–9, sometimes aborted (1–7), dark brown or brown mottled with paler irregular lines, semi-ellipsoid, 1.4–2.7 × 0.8–1.3 cm, rounded at both ends, with a yellow to orangish yellow fleshy pulp.

**Figure 1.**
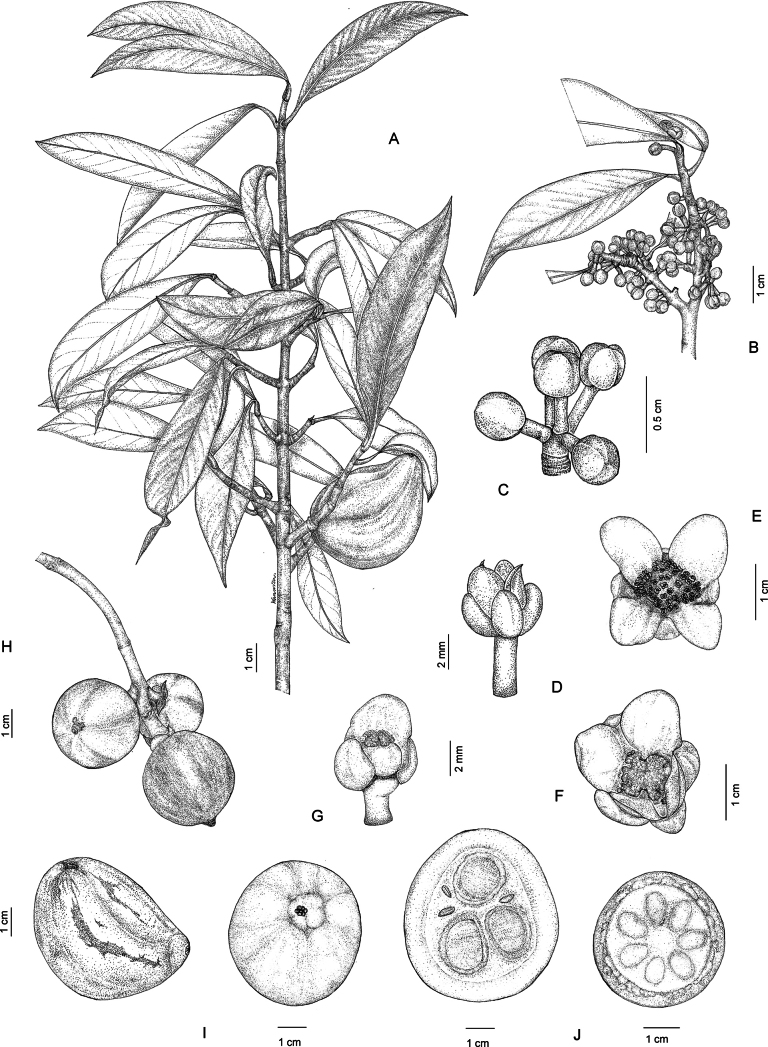
*Garcinia cowa* var. *cowa*. **A**. Branchlets with leaves and fruit; **B**. Branchlets with leaves and inflorescences bearing staminate flower buds; **C**. Inflorescence with staminate flower buds; **D**. Staminate flower in side view; **E**. Staminate flower in top view; **F**. Pistillate flower in top view; **G**. Pistillate flower in side view, with some sepals and petals removed; **H, I**. Fruits; **J**. Fruits in transverse section showing seeds with fleshy pulp. Photo: Drawn by Wanwisa Bhuchaisri.

**Figure 2.**
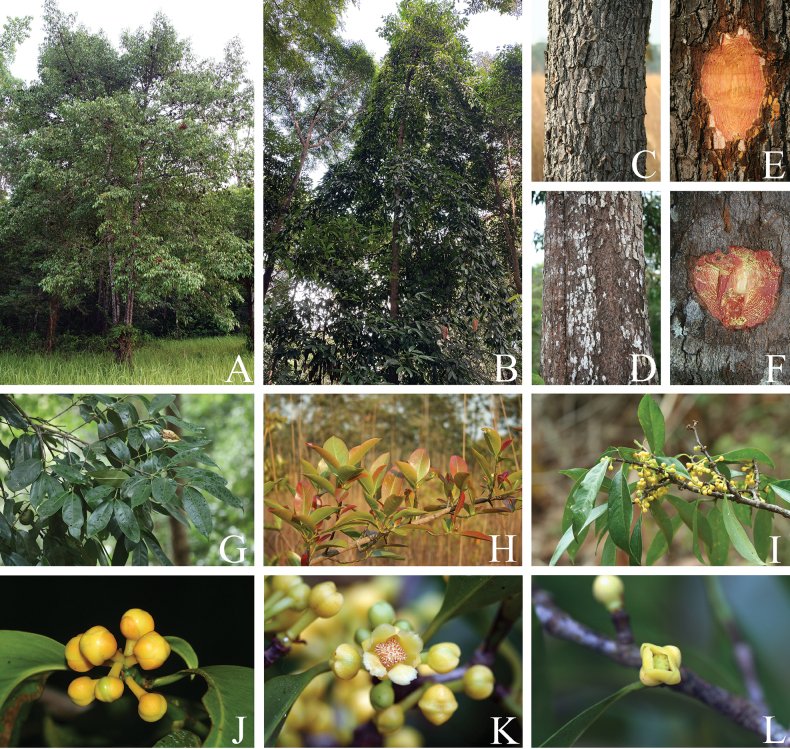
*Garcinia cowa* var. *cowa*. **A, B**. Habit and habitats; **C, D**. Bark; **E, F**. Slashed bark with yellow exudate; **G**. Branchlets with leaves; **H**. Branchlets with young leaves; **I**. Branchlets with leaves and inflorescences bearing staminate flower buds; **J**. Inflorescence with staminate flower buds; **K**. Inflorescence with open staminate flower and flower buds; **L**. Branchlets with open pistillate flower. Photos: Chatchai Ngernsaengsaruay.

**Figure 3.**
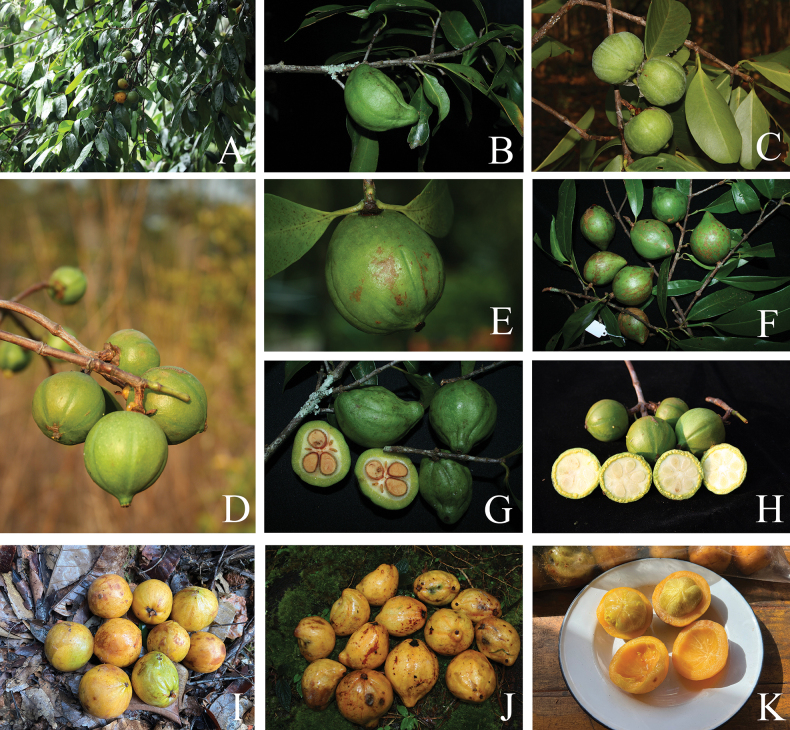
*Garcinia cowa* var. *cowa*. **A**. Branchlets with leaves and fruits; **B–F**. Branchlets with mature and ripe fruits; **G, H**. Fruits and transverse sections of fruits showing yellow exudate and seeds; **I, J**. Ripe fruits; **K**. Transverse sections of fruits showing pericarp and seeds with yellow fleshy pulp. Photos: Chatchai Ngernsaengsaruay.

##### Distribution.

**Figure 4.**
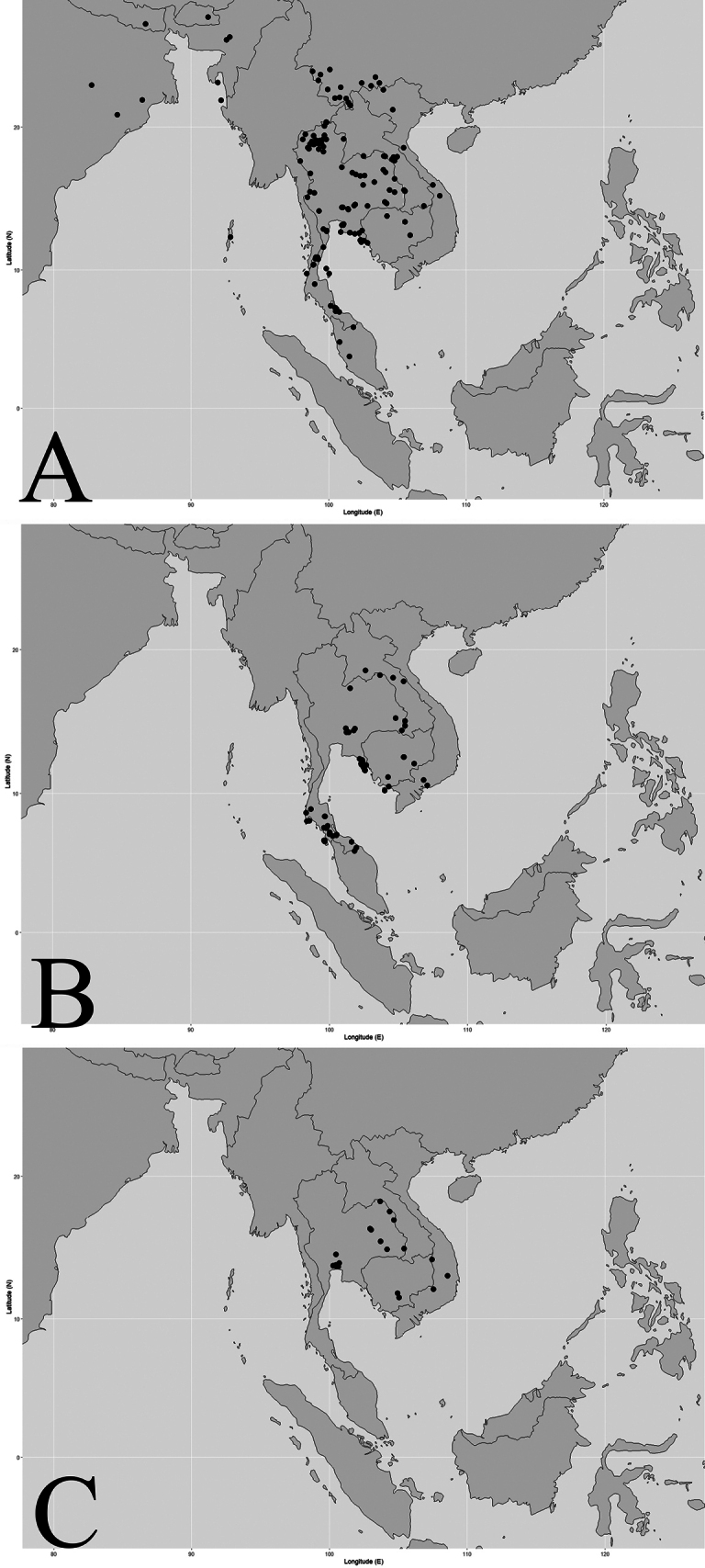
Distribution of three Thai *Garcinia* species. **A**. *Garcinia cowa* var. *cowa* is widely distributed from India to China and Peninsular Malaysia; in Thailand, it occurs naturally throughout all seven floristic regions; **B**. *Garcinia oliveri* is widely distributed from Indo-China to Peninsular Malaysia; in Thailand, it occurs in the north-eastern, eastern, central, south-eastern, and peninsular regions; **C**. *Garcinia schomburgkiana* is distributed from Indo-China to Thailand; in Thailand, it occurs naturally in the northern, north-eastern, eastern, and central regions. Maps: Pichet Chanton and Chatchai Ngernsaengsaruay.

India (Assam, Andaman and Nicobar Islands), Nepal, Bhutan, Bangladesh, Myanmar, China (West and South Yunnan), Vietnam, Laos, Cambodia, Thailand, Peninsular Malaysia (Fig. 4A).

##### Distribution in Thailand.

It is found in all floristic regions. **Northern**: Mae Hong Son, Chiang Mai, Chiang Rai, Phayao, Nan, Lamphun, Lampang, Tak, Phitsanulok; **North-Eastern**: Loei, Nong Khai, Bueng Kan, Sakon Nakhon, Mukdahan, Maha Sarakham, Khon Kaen; **Eastern**: Nakhon Ratchasima, Buri Ram, Yasothon, Si Sa Ket, Ubon Ratchathani; **South-Western**: Kanchanaburi, Phetchaburi, Prachuap Khiri Khan; **Central**: Saraburi, Nakhon Nayok; **South-Eastern**: Chon Buri, Rayong, Chanthaburi, Trat; **Peninsular**: Chumphon, Ranong, Surat Thani, Songkhla, Narathiwat (Fig. 4A).

##### Habitat and ecology.

This species is found in a wide variety of habitats, including tropical lowland evergreen rain forests, dry evergreen forests, coastal dry evergreen forests, peat swamp forests, beach forests, deciduous dipterocarp forests, pine–deciduous dipterocarp forests, mixed deciduous forests, mixed deciduous forests on limestone hills, lower montane rain forests, lower montane pine–oak forests, forest edges, and secondary forests. It is often found along streams, at elevations from near sea level up to 1,500 m a.m.s.l.

##### Phenology.

Flowering and fruiting occur more than once, nearly throughout the year; flowering peaks from November to April, and fruiting peaks from March to August.

##### Conservation status.

*Garcinia cowa* var. *cowa* is widely distributed from India to China and Peninsular Malaysia. It has been recorded from numerous localities and has a large Extent of Occurrence (EOO) of 229,508,285.78 km^2^ and an Area of Occupancy (AOO) of 712 km^2^. In Thailand, it occurs naturally throughout all seven floristic regions, with an EOO of 900,464.98 km^2^ and an AOO of 440 km^2^. Given its wide distribution and the abundance of localities, the species faces no significant threat of extinction. We therefore assess its conservation status as Least Concern (LC), in agreement with Deepu and Geethakumary (2020).

##### Etymology.

The specific epithet of *Garcinia cowa* is derived from the local Hindi name “Cowa”, which is a vernacular name used for this species in India (Roxburgh 1832; Planchon and Triana 1860).

##### Vernacular names.

Ka muang (กะมวง) (Chumphon, from the specimen *A. F. G. Kerr 11624*); Ka muang sai (กะมวงทราย) (Chumphon, from the specimen *A. F. G. Kerr 11474*); Ka-ni (กานิ) (Malay-Narathiwat, reported by Pooma and Suddee 2014); Kwa (กวะ) (Karen-Kanchanaburi, from the specimen *A. F. G. Kerr 10317*); **Cha muang** (ชะมวง) (General, Chon Buri, from the specimen *D. J. Collins 969*, Trat, from the specimen *C. Phengklai et al. 13144*); Nguan lueang (ง้วนเหลือง) (Loei, from the specimen *K. Suvatabandhu 220*); Tong mang (โตงมาง) (Sakon Nakhon, from the specimen *W. McClatchey WCM3470*); Nam nong (น้ำนอง) (Kanchanaburi, from the specimen *K. Bunchuai 37*); Bong nang (บงนั่ง) (Sakon Nakhon, from the specimen *P. Suvarnakoses 2035*); Ma nguang (มะงวง) (Phetchaburi, from the specimen *A. F. G. Kerr 20613*); Ma mong (มะโมง) (Saraburi, from the specimen *Unknown 10* at BKF12125); Ma pong (มะป่อง) (Chiang Mai, from the specimen *T. Santisuk 6642*); Mong (มง) (Ranong, from the specimen *A. F. G. Kerr 16349*; Saraburi, from the specimen *A. Wanaraks 77*); Mong (โมง) (Si Sa Ket, from the specimens *A. F. G. Kerr 8324* and *P. Suvarnakoses 1486*); Muang (มวง) (Surat Thani, from the specimen *A. F. G. Kerr 18221*); Muang som (มวงส้ม) (Nakhon Si Thammarat, reported by Pooma and Suddee 2014); Yang nok (ยางนก) (Trat, from the specimen *C. Phengklai et al. 13361*); Som kung yai (ส้มกุ้งใหญ่) (Khon Kaen, from the specimen *Prakob 6* at BKF186121); Som nguang (ส้มงวง) (Saraburi, from the specimen *Unknown 10* at BKF12125); Som pong (ส้มป่อง) (Phayao, from the specimen *Put 3984*); Som mong (ส้มโมง) (Ubon Ratchathani, from the specimen *P. Puudjaa 1443*); Som muang (ส้มมวง) (Buri Ram, from the specimen *S. Suksakorn 981*; Sakon Nakhon, from the specimen *W. McClatchey WCM3470*); Mak mok (หมากโมก) (Udon Thani, reported by Pooma and Suddee 2014); Mak mong (หมากโมง) (Nong Khai, from the specimen *K. Bunchuai 1829*); Mak mong (หมักหมง) (Phitsanulok, from the specimen *A. F. G. Kerr 5841*).

##### Uses.

*Garcinia cowa* var. *cowa* is cultivated for its young leaves, which have a naturally sour taste. These leaves are used in a variety of boiled dishes, such as red curry with pork and *cha muang* leaves, as well as chicken, pork rib, braised pork leg, or beef soups. Young leaves can also be eaten fresh as a vegetable. The fleshy pulp surrounding the seeds of the ripe fruits is edible and has a sweet-and-sour taste (from the author’s observations).

##### Lectotypifications.

*Oxycarpus cochinchinensis* Lour. was originally named by de Loureiro (1790) and later transferred to the genus *Garcinia* by Choisy (1823). In doing so, Choisy cited the type locality as Cochinchina and noted that the species grows in both cultivated and uncultivated conditions (“Habitat: tam cultus, quam incultus in Cochinchinâ”). However, he did not indicate a type, nor did he provide a collector number or specify the herbarium where the material was deposited. We have located two sheets of the specimen *J. de Loureiro* without a number (*s.n*.) from Vietnam (southern Cochinchina), currently housed at BM [BM000611621, BM000611622]. According to Article 9.6 of the International Code of Nomenclature for algae, fungi, and plants (ICN) (Turland et al. 2018), these specimens constitute syntypes. Of these, specimen BM000611621 is better preserved and more complete than the other and is therefore designated here as the lectotype, with BM000611622 designated as an isolectotype, following Arts. 9.3 and 9.12 of the ICN (Turland et al. 2018).

*Garcinia nigrolineata* was described by Anderson (1874), who cited three gatherings collected from Malacca: *Griffith* (Kew Distrib. *854*) and *Maingay* (Kew Distrib. *152* and *162*). However, Anderson did not indicate a type, nor did he specify the herbaria where the specimens were deposited. We traced the cited material from Malacca as follows: *W. Griffith 854* (Distributed at Kew, 1861–1862) is located at CAL [CAL0000005801], GH [GH00067491], K [K000677662], L [L.2417126], P [P04701603], and W [W-0073380 (Marinho 2017)]; *A. C. Maingay 152* (Distributed at Kew, 1871) is found at BM [BM000611778], CAL [CAL0000005800], GH [GH00067492], K [K000677660, K000677661], and L [L.2417125]; *A. C. Maingay 162* (Distributed at Kew, 1871) is preserved at CAL [CAL0000005799]. Following Art. 9.6 of the ICN (Turland et al. 2018), these specimens constitute syntypes. Among them, the specimen *W. Griffith 854* (Distributed at Kew, 1861–1862) at K [K000677662] is in the best condition and clearly exhibits the diagnostic characters for the species. It is therefore selected here as the lectotype, following Arts. 9.3 and 9.12 of the ICN (Turland et al. 2018).

*Garcinia loureiroi* was described by Pierre (1883), who cited two gatherings: *Herb. Pierre 420* and *669*. In the protologue, the species was noted as being cultivated throughout the provinces of Lower Cochinchina and Cambodia (“Habitat: Cette espèce est cultivée dans toutes les provinces de la Basse-Cochinchine et du Cambodge”). We traced the specimens cited in the protologue to the following: *J. B. L. Pierre 420*, cultivated near the village of Cây bé in southern Cochinchina (“Culta ad pagum Cây bé austro gallicae Cochinchinae”), represented at P by specimens [P05061498, P05061501, P05061502, P05061503]; and *J. B. L. Pierre 699*, also cultivated in Cochinchina. Upon examination, the specimens cited as number “*669*” in the original description correspond in all respects to the specimen labelled “*699*,” and no distinct gathering corresponding to “*669*” was located. We therefore interpret the citation of “*Herb. Pierre 669*” in the protologue as a transcription error and correct it to “*J. B. L. Pierre 699*.” Specimens under the collection number *699* represent multiple gatherings differing in locality and date and can be grouped as follows:

Group A: *Harmand 570* (*Herb. L. Pierre 699*), cultivated near the village of Cây bé in southern Cochinchina (“Culta ad pagum Cây bé austro gallicae Cochinchinae”), collected in June 1876. Specimen at: P [P05061508].

Group B: *J. B. L. Pierre 699*, cultivated in the Saigon Botanical Garden (in the garden of Cochinchina (“Culta in hort. bot. Saigoneum, in horti Cochinchinae”), collected in July 1877. Specimens at: P [P05061423, P05061511].

Group C: *J. B. L. Pierre 699*, same locality as above, collected in September 1877. Specimens at: P [P05061495, P05061496, P05061507].

Group D: *J. B. L. Pierre 699*, same locality, collected in May 1879. Specimen at: P [P05061509].

As Pierre did not indicate a type nor specify the herbarium housing the original material, all cited specimens are considered syntypes, following Art. 9.6 of the ICN (Turland et al. 2018). We designated here the specimen *J. B. L. Pierre 420* at P [P05061502] as the lectotype, as it is the most complete and best-preserved specimen among the syntypes, following Art. 9.3 of the ICN (Turland et al. 2018). The remaining specimens under the same gathering [P05061498, P05061501, P05061503] are designated as isolectotypes, in accordance with Art. 9.12 of the ICN (Turland et al. 2018).

*Garcinia fusca* was named by Pierre (1883), citing only one collection: *Herb. Pierre 3622*, reported as inhabiting the banks of the Saigon River and the Angor Province in Cambodia (“Cette espèce habite les rives du fleuve de Saïgon et la province d’Angkor dansle Cambodge”). However, upon examination, we found that the materials are morphologically similar, even though the specimens under this collection number differ in both locality and collection date and can be separated into four distinct groups:

Group A: *J. B. L. Pierre 3622* from the banks of the Saigon River, Vietnam (“ad flumen Saigon gallicae austro Cochinchinae”), collected on 18 April 1866. Specimens at: BM [BM001191365], K [K000742484], L [L0700338, L0700339], and S [S11-34587].

Group B: *J. B. L. Pierre 3622* from Cai Cong, Tây Ninh Province, Vietnam (“ad Cai Cong in prov. Tây Ninh”), also collected on 18 April 1866. Specimens at: K [K000677696], P [P04701639, P04701672, P04701676, P04701677], and U [U1208222]

Group C: *J. B. L. Pierre 3622* from forests near Cai Cong, Tây Ninh, Vietnam (“in sylvis ad Cai Cong in prov. Tây Ninh gallicae austro Cochinchinae”), collected in June 1866. Specimen at: P [P04701673]

Group D: *Harmand* (*Herb. L. Pierre 3622*) from Angkor, Siem Reap, Cambodia, collected in September 1873. Specimen at: P [P04701644].

Pierre did not indicate a type or specify the herbaria where the specimens were deposited. Following Art. 9.6 of the ICN (Turland et al. 2018), these specimens are considered syntypes. Therefore, the specimen *J. B. L. Pierre 3622* of Group A at K [K000742484] is designated here as the lectotype, with the specimens at BM [BM001191365], L [L0700338, L0700339], and S [S11-34587] designated as isolectotypes, following Arts. 9.3 and 9.12 of the ICN (Turland et al. 2018).

*Garcinia kunstleri* was described by King (1890) based on specimens collected by King’s Collector, Scortechini, and Wray from low elevations in Perak. King did not indicate a type, nor did he provide collector numbers or specify the herbaria in which the specimens were housed. We have located the following specimens from Perak: *King’s Collector 2218* at CAL [CAL0000005874], *King’s Collector 4317* at G [G00726360], *King’s Collector 7007* at CAL [CAL0000005876], *King’s Collector 8196* at CAL [CAL0000005875, CAL0000005880], *King’s Collector 8302* at CAL [CAL0000005883], G [G00726359], and US [US02961153], *King’s Collector 8809* at CAL [CAL0000005877], *King’s Collector 10208* at CAL [CAL0000005881, CAL0000005882], *B. Scortechini 271* at CAL [CAL0000005869], G [G00726398], and SING [SING0063105], *B. Scortechini 1852* at SING [SING0063106], *B. Scortechini 1388* at CAL [CAL0000005871] and G [G00726394], *B. Scortechini s.n*. at SING [SING0063107], *L. Wray 328* at CAL [CAL0000005872, CAL0000005873], and *L. Wray 828* at SING [SING0063122, SING0063123, SING0063124]. Following Art. 9.6 of ICN (Turland et al. 2018), all these specimens are treated as syntypes. George King served as Superintendent of Calcutta Botanic Gardens (1871–1898) and as Director of the Botanical Survey of India (1891–1898) (Stafleu and Cowan 1979). We hereby designate the specimen *L. Wray 328* at CAL [CAL0000005872] as the lectotype, as it is the most complete and best-preserved among the syntypes, in accordance with Art. 9.3 of the ICN (Turland et al. 2018). The duplicate specimen from the same gathering at CAL [CAL0000005873] is designated as an isolectotype, following Art. 9.12 of the ICN (Turland et al. 2018).

*Garcinia plena* was described by Craib (1924) based on the specimen *A. F. G. Kerr 5007*, collected from Ban Tiu, Nan Province, at an elevation of approximately 1,100 m a.m.s.l. Craib did not select a holotype, nor did he specify the herbarium in which the specimen was housed. However, we have located three sheets of the specimen *A. F. G. Kerr 5007* at BK [BK257947], BM [BM000611604], and K [K000677703]. Following Art. 9.6 of ICN (Turland et al. 2018), these are treated as syntypes. William Grant Craib (1882–1933), a British botanist affiliated with Aberdeen and Kew (Stafleu and Cowan 1976), likely had access to the material at K. Therefore, the specimen at K [K000677703] is designated here as the lectotype, with the specimens at BK [BK257947] and BM [BM000611604] designated as isolectotypes, in accordance with Arts. 9.3 and 9.12 of the ICN (Turland et al. 2018).

##### Notes.

The morphological variability observed in the leaves and fruits of *Garcinia cowa* var. *cowa* and the previously recognized taxa further supports their synonymization. Examination of herbarium specimens, together with protologue descriptions and previous studies (e.g., de Loureiro 1790; Anderson 1874; Kurz 1877; Pierre 1883; Vesque 1889, 1893; King 1890; Pitard 1910; Craib 1924; Gagnepain 1943; Maheshwari 1964; Whitmore 1973; Long 1984; Singh 1993; Li et al. 2007; Mohanan et al. 2023), shows that leaf shape and size are highly variable across these taxa. Fruits also exhibit a wide range of forms, being shallowly or weakly 6–9-lobed, or sometimes unlobed, with or without a short, thick apical beak. Such variation in both vegetative and reproductive characters overlaps extensively among *G. cowa* var. *cowa*, *G. cochinchinensis*, *G. fusca*, *G. nigrolineata*, and *G. plena*, making them morphologically indistinguishable.

Given this extensive overlap, these four taxa cannot be reliably separated based on morphology. Therefore, consistent with previous studies and our own observations, we treat *G. cochinchinensis*, *G. fusca*, *G. nigrolineata*, and *G. plena* as new synonyms of *G. cowa* var. *cowa*. This treatment reflects the morphological continuum observed in these taxa and aligns with earlier taxonomic work, helping to reduce ambiguity in species identification.

*Garcinia kydia* Roxb. has been reduced to *G. cowa* Roxb. ex Choisy var. kydia (Roxb.) Shameer and N. Mohanan (Mohanan et al. 2023). However, following Mohanan et al. (2023), the correct authorship of *G. cowa* is Roxb. ex Choisy, not Roxb. ex DC. This is because *G. cowa* Roxb. ex Choisy, published earlier (Choisy 1823), is the accepted name, whereas *G. cowa* Roxb. ex DC., published later (de Candolle 1824), is illegitimate under the ICN. *Garcinia cowa* var. *cowa* differs from *G. cowa* var. kydia in having pistillate inflorescences with 2–3 flowers and fruits that are non-mamillate and grooved from the base to the apex. In contrast, *G. cowa* var. kydia has solitary pistillate flowers and fruits that are mamillate and grooved only toward the apex (Mohanan et al. 2023).

*Garcinia kunstleri* was treated as a synonym of *G. nigrolineata* by Kochummen and Whitmore (1973).

According to Pooma and Suddee (2014), the local name of *Garcinia fusca* is Ma dan pa (มะดันป่า) in Maha Sarakham Province. However, we found that the specimen *T. Smitinand 10441* at BKF [BKF36151] collected from Maha Sarakham Province, was misidentified as *G. fusca*, whereas the correct identification is *G. schomburgkiana*.

##### Additional specimens examined.

**Thailand. Northern**: • Mae Hong Son [Ban Nong Khao Klang, Mueang Mae Hong Son District, fl., 25 Mar 2006, *S. Watthana 1883* (CMUB, QBG)]; • Chiang Mai [Doi Suthep, fl., 17 Apr 1910, *A. F. G. Kerr 1124* (BM, K [K003964730, K003964731], TCD [TCD0010441]); • ibid., fr., 9 May 1911, *A. F. G. Kerr 1828* (BM, E [E00839766], K [K003964729], P [P04701449], TCD [TCD0010440]); • ibid., fr., 15 Nov 1914, *A. F. G. Kerr 3464* (BM, K [K003964711]); • ibid., sterile, 17 Dec 1958 (as *Garcinia* sp.), *T. Sørensen, K. Larsen & B. Hansen 6481* (BKF, C); • ibid., fr., 17 Jul 1968 (as *Garcinia* sp., *G. cf. fusca*), *K. Larsen, T. Santisuk & E. Warncke 2595* (AAU, BKF, E [E00839765], K [K003964715], P [P05062016]); • ibid., fr., 14 Aug 1968 (as *Garcinia* sp.), *S. Phengnaren 215* (BKF); • Pha Lat, Doi Suthep, fr., 25 Jul 1987 (as *G. speciosa*), *J. F. Maxwell 87-704* (BKF, L [L.2416705]); • Montha Than Waterfall, Doi Suthep, ♂ fl., 6 Feb 1988, *J. F. Maxwell 88-135* (AAU, BKF, L [L.2403055]); • Pha Ngoep, Doi Suthep, ♀ fl., 1 Dec 1988, *J. F. Maxwell 88-1362* (AAU, BKF, L [L.2403056]); • Doi Suthep-Pui National Park, fr., 7 Mar 1992, *J. F. Maxwell 92-67* (CMUB, P [P04701436]); • ibid., ♂ fl., 8 Apr 1992, *J. F. Maxwell 92-133* (CMUB); • ibid., sterile, 18 May 1992, *T. Santisuk s.n*. (BKF118370); • ibid., ♂ fl., 15 Oct 1992, *J. F. Maxwell 92-620* (BKF, CMUB, L [L.3806466], P [P04701433]); • ibid., fl., 20 Feb 2004 (as *G. cf. parvifolia*), *P. Wilkie et al. PW436* (BKF); • Chang Khian Village, Doi Suthep-Pui National Park, fr., 10 May 1995, *S. Kopachon S120* (CMUB); • Khun Wang, ♀ fl., 5 Apr 1978, *T. Smitinand s.n*. (BKF072733); • ibid., fl., 5 Apr 1978 (as *Garcinia* sp.; H. Toyama det. as *G. fusca*), *T. Smitinand s.n*. (BKF073225); • Doi Inthanon, Chom Thong District, fl., 16 Feb 1998 (as *G. speciosa*), *F. Konta et al. 4324* (BKF); • Pha Mon, Doi Inthanon, fl., 16 Feb 1998 (as *G. speciosa*), *C. Phengklai et al. 11036* (BKF); • Doi Inthanon National Park, fr., 3 Aug 2003 (as *Garcinia* sp.), *T. Wongprasert & S. Khao-iam 038-39* (BKF); • Ban Angka Noi, Doi Inthanon National Park, fr., 4 Aug 2003 (as *Garcinia* sp.), *T. Wongprasert & S. Khao-iam 038-40* (BKF); • Mae Klang Luang Village, Doi Inthanon National Park, fr., 4 May 2010, *P. Georgiadis 278* (CMUB, L [L.2058950], P [P00931054]); • Mae Taeng District, ♂ fl., 10 Jan 1988 (as *G. cf. speciosa*), *T. Santisuk 6642* (BKF); • Doi Chiang Dao Wildlife Sanctuary, Chiang Dao District, ♂ fl., 10 Oct 1995, *J. F. Maxwell 95-880* (BKF, CMUB, L [L.3881945]); • Thep Sadet Subdistrict, Doi Saket District, ♂ fl., 23 Jul 1996 (as *G. speciosa*), *J. F. Maxwell 96-999* (CMUB); • Huai Mae Mae, Mae Rim District, fl., 23 Mar 1999 (as *Garcinia* sp.), *P. Rittisuntorn 16* (PSU); • Ban Phra Bath Si Roi, Sa Luang Subdistrict, Mae Rim District, fl., 30 Mar 2007 (as *Garcinia* sp.), *W. Pongamornkul 1912* (QBG); • Queen Sirikit Botanical Garden, Mae Rim District, sterile, 23 Jan 2013, *J. A. van der Scheur et al. 90* (L [L.2065861]); • ibid., fr., 1 Jun 2016 (as *Garcinia* sp.), *C. Glamwaewwong 138/60* (QBG); • Mae Kam Pong Waterfall, Mae On District, ♂ fl., 5 Apr 2004, *J. F. Maxwell 04-184* (BKF, CMUB, L [L.3812990, L.3878614]); • ibid., fl., 25 Mar 2012, *M. Norsangsri & S. Mattapha 9181* (AAU, QBG); • Ban Lao Wa, Wiang Haeng District, fr., 18 Jul 2001 (as *Garcinia* sp.), *M. Norsaengsri 1578* (QBG); • Pha Dam Waterfall, Mae Win Subdistrict, Mae Wang District, ♀ fl., 22 Apr 2004, *J. F. Maxwell 04-231* (CMUB, L [L.3812988]); • Huai Kaeo Subdistrict, Mae On District, fr., 15 Jul 2005 (as *G. speciosa*), *J. F. Maxwell 05-437* (BKF, CMUB); • ibid., fr., 7 Sep 2011 (as *Garcinia* sp.), *R. Pooma et al. 7792* (BKF); • Huai Hong Khrai, fr., 13 Aug 2007 (as *G. succifolia*), *P. Klomsakul 7, 8* (BKF); • Ban Pok Nai, fr., 23 Apr 2008 (as *Garcinia* sp.), *Pimsiri PN001* (QBG); • Samoeng District, fr., 24 Jun 2008 (as *Garcinia* sp.), *K. Jatupol 08-229* (QBG, SING)]; • Chiang Rai [Ku Tan Mountain, seed, 30 Jan 1970, *S. Sutheesorn (Sutisorn) 1510* (BK); • Doi Luang National Park, Mae Suai District, fr., 6 Jun 1998, *P. Sidisunthorn & S. Gardner 2723* (CMUB); • Khun Chae National Park, Wiang Pa Pao District, ♂ fl., 2 Apr 1998 (as *G. propinqua*), *J. F. Maxwell 98-364* (CMUB, L [L.3806395]); • Doi Tung, Mae Sai District, ♀ fl., 7 Apr 2006, *J. F. Maxwell 06-274* (CMUB, L [L.3878632]); • Ban Pha Mi, Mae Sai District, fl., 22 Mar 2011 (as *G. merguensis*), *M. Norsangsri & N. Tathana 7844* (BKF); • Pa Kook Village, Mae Sai District, fl., 3 Feb 2012, *S. Laphookhieo 1* (CMUB)]; • Phayao [Mueang Payao District, fl., 12 Jul 1931 (as *Garcinia* sp.), *Put 3984* (BM, K [K003964725]); • Champa Thong Waterfall, Doi Luang National Park, Mueang District, fr., 10 Aug 1997 (as G. speciosa), *P. Sidisunthorn & S. Gardner 2289* (CMUB)]; • Nan [Doi Phu Kha National Park, ♂ fl., 18 Mar 1999 (as *Garcinia* sp.), *P. Srisanga 522* (BKF, CMUB, QBG)]; • Lamphun [Doi Khun Tan National Park, Mae Tha District, fr., 29 Aug 1993, *J. F. Maxwell 93-1006* (BKF, CMUB)]; • Lampang [Mae Chang, fl., 22 Jan 1926 (as *Garcinia* sp., *G. gracilis*), *Winit 1579* (BK, BKF K [K003964699]); • Chae Son National Park, ♂ fl., 28 Mar 1996, *J. F. Maxwell 96-436* (BKF, CMUB, L [L.3881940]); • Wang Nuea District, ♂ fl., 26 Mar 1997 (as *G. hanburyi*), *J. F. Maxwell 97-247* (BKF, CMUB); • Chae Hom District, ♂ fl., 5 Nov 2000, *M. Panatkool 417* (CMUB)]; • Tak [Mae Sot District, fr., 30 May 1973 (as *Garcinia* sp.), *R. Geesink et al. 5553* (C); • Tha Song Yang District, fl., 19 Jan 1995 (as *Garcinia* sp.), *R. Pooma 1001* (BKF, CMUB)]; • Phitsanulok [Nakhon Thai District, fr., 15 Apr 1922 (as *Garcinia* sp.), *A. F. G. Kerr 5841* (BK, BM, K [K003964717])]; **North-Eastern**: • Loei [Phu Kradueng, fl., 6 Apr 1948 (as *G. plena*; H. Toyama det. as *G. fusca*), *K. Suvatabandhu 220* (BK)]; • Nong Khai [Dong Chom Phu, Phon Phisai District, fr., 9 May 1969, *K. Bunchuai 1829* (BKF)]; • Bueng Kan [Ban Tong, Bueng Khong Long Non-Hunting Area, Bueng Khong Long District, fl., 22 May 2004 (as *Garcinia* sp.), *R. Pooma et al. 4248* (AAU, BKF)]; • Sakon Nakhon [Phu Phan National Park, fl., 9 Dec 1962 (as *G. gracilis*), *P. Suvarnakoses 2035* (BKF, K [K003964714]); • ibid., fl., 14 Dec 1982 (as *Garcinia* sp.; H. Toyama det. as *G. fusca*), *H. Koyama et al. T-31000* (BKF); • Ban Nong Pan, Kham Bo Subdistrict, Waritchaphum District, sterile, 17 Feb 2006 (as *G. speciosa*), *W. McClatchey WCM3470* (BKF)]; • Mukdahan [Phu Pha Thoep National Park, fr., 11 May 1997 (as *Garcinia* sp.; H. Toyama det. as *G. cf. fusca*), *R. Pooma 1643* (BKF, CMUB)]; • Maha Sarakham [Walai Rukhavej Botanical Research Institute, Na Dun Distict, fl., 13 Jan 2009 (as *G. nigrolineata*), *N. Norsaengsri 4671* (QBG)]; • Khon Kaen [Locality unspecified, sterile, 18 Aug 1968 (as *G. lanessanii*), *Prakob 6* (BKF186121); • Hin Chang Si, Nam Phong District, fl., 18 Jan 2004, *ATRC Staff 24* (AAU); • Khok Phu Ta Ka, Phu Wiang District, fl., 4 Nov 2006, *P. Krachai 327* (AAU); • Phu Han–Phu Ra-ngam Forest Park, Chonnabot District, fl., 30 Oct 2007(as *Garcinia* sp.), *M. Norsaengsri 2833* (QBG); • Phu Pha Man National Park, fr., 12 May 2010 (as *Garcinia* sp.), *M. Norsaengsri 6686* (QBG)]; **Eastern**: • Nakhon Ratchasima [Pak Thong Chai District, fl., 26 Dec 1923 (as *Garcinia* sp.), *A. F. G. Kerr 8116* (BK, BM, K [K003964721]); • Wang Nam Khiao District, fl., 26 Feb 1963 (as *G. speciosa*; P. F. Stevens det. as *G. cf. celebica*), *Adisai 414* (BK); • Khao Yai National Park, Pak Chong District, ♂ fl., 15 Feb 2003, *A. Boonkongchart 201* (BKF, CMUB, L [L.3813581, L.3813582])]; • Buri Ram [Nang Rong District, Mar 1920 (as *Garcinia* sp.), *S. Suksakorn 981* (BKF13506)]; • Yasothon [Kham Khuen Kaeo District, fl., Mar 1982 (as *Garcinia* sp.; H. Toyama det. as *G. fusca*), *T. Smitinand s.n*. (BKF98547)]; • Si Sa Ket [Huai Nuea, Khukhan District, fl., 20 Jan 1924 (as *Garcinia* sp.), *A. F. G. Kerr 8324* (BK, BM, C, K [K003964713]); • Thung Nong Ma Saeo, Kanthararom District, ♂ fl., 15 Feb 1959, *P. Suvarnakoses 1486* (BKF)]; • Ubon Ratchathani [Ban Nong Khun, Muang Sam Sip District, sterile, 11 Dec 1982 (as *Embelia subcoriacea*), *H. Koyama et al. T-30822* (BKF); • Phu Pang, Buntharik District, fr., 14 May 2005 (as *Garcinia* sp.), *P. Puudjaa 1443* (BKF); • Dong Na Tham Forest, Pha Taem National Park, Khong Chiam District, ♀ fl., 28 Feb 2007, *S. Suddee et al. 3070* (BKF)]; **South-Western**: • Kanchanaburi [Wangka Subdistrict, Sangkhla Buri District, fl., 26 Jan 1926 (as *Garcinia* sp.), *A. F. G. Kerr 10317* (BK, BM, K [K003964698]); • Locality unspecified, ♀ fl. & fr., 17 Feb 1960, *K. Bunchuai 37* (BKF, C, E [E00839763], K [K003964716], P [P05062026]); • Khao Ngi Yai, E. of Sangkhla Buri District, fr., 4 Apr 1968, *C. F. van Beusekom & C. Phengkhlai 323* (AAU, BKF, C, K [K003964701], P [P05062034]; • Thung Yai Naresuan Wildlife Sanctuary, Lai Wo District, ♂ fl., 8 Nov 2001, *Martin van de Bult 487* (BKF, CMUB, L [L.3813641])]; • Phetchaburi [Thung Luang, fl., 9 Nov 1931 (as *Garcinia* sp.), *A. F. G. Kerr 20613* (BM, K [K003964726]); • Kaeng Krachan National Park, Kaeng Krachan District, fr., 10 May 2005, *D. J. Middleton et al. 3356* (BKF, E [E00219217]); • Tha Yang District, fl., 16 Feb 2006, *D. J. Middleton et al. 3713* (BKF, E [E00261365])]; • Prachuap Khiri Khan [Huai Yang National Park, Thap Sakae District, ♀ fl. & fr., 25 Jan 2004 (as *Garcinia* sp.), *D. J. Middleton et al. 2501* (BKF, E [E00351636], K); • ibid., ♂ fl., 25 Jan 2004 (as *Garcinia* sp.), *D. J. Middleton et al. 2502* (BKF, E [E00351628]); • Namtok Huai Yang National Park, fr., 31 Mar 1999 (as *Garcinia* sp.), *K. Phattharahirankanok 34* (BKF)]; **Central**: • Saraburi [Ban Huai Haeng, fr., 24 Mar 1927 (as *Garcinia* sp.), *A. Wanaraks 77* (BK, K [K003964718]); • Phu Khae, fl., 30 Jan 1948, (as *Garcinia* sp.), *Unknown 10* (BKF12125); • Sam Lan Forest, Mueang District, fr., 15 Jul 1975, *J. F. Maxwell 75-691* (AAU, BK, L [L.2403063])]; • Nakhon Nayok [Khao Yai National Park, Mueang Nakhon Nayok District, fl., 20 Feb 2003, *P. Charoenchai & S. Poompuang 374* (BK, CMUB); • Khao Yai National Park, fr., 27 Jun 2001, *J. F. Maxwell 01-330* (BKF, CMUB, L [L.3813631])]; **South-Eastern**: • Chon Buri [Si Racha Forest, fl., Feb 1914 (as *Garcinia* sp.), *D. J. Collins 371* (K [K003964724]); • Khao Chalat, Si Racha District, fl., 8 Mar 1924 (as *Garcinia* sp.), *D. J. Collins 969* (BK, K [K003964704]); • Nong Takrum, Sriracha District, fl., 11 Jan 1928 (as *Garcinia* sp.), *D. J. Collins 1951* (BK, BM, K [K003964703]); • Khao Din near Si Racha District, fl., 23 Feb 1928 (as *Garcinia* sp.), *D. J. Collins 2046* (BK, K); • ibid., fr., 25 Mar 1928 (as *Garcinia* sp.), *D. J. Collins 2064* (BK, K [K003964702]); • Thung Prong, Sattahip District, fl., 10 Feb 1974, *J. F. Maxwell 74-133* (AAU, BK); • Khao Khiao, Si Racha District, ♂ fl., 4 Mar 1975 (as *G. speciosa*), *J. F. Maxwell 75-186* (AAU, BK)]; • Rayong [Chak Phong Subdistrict, Klaeng District, ♂ fl., 29 May 2002, *K. Kertsawang 38* (QBG); • Rayong Botanical Garden, fl., 14 May 2015 (as *G. nigrolineata*), *C. Maknoi 7762* (QBG, this specimen contains two species); • ibid, fr., 14 May 2015 (as *G. nigrolineata*), *C. Maknoi 7780* (QBG)]; • Chanthaburi [Makham District, fr., 3 Jun 1965 (as *Garcinia* sp.), *C. Phengklai 1083* (BKF); • Pong Nam Ron District, sterile, 16 Feb 1967 (as *Garcinia* sp.), *T. Smitinand 10185* (BKF); • Khung Kraben, Tha Mai District, fr., 27 May 1994, *P. Puudjaa 141* (BKF)]; • Trat [Ko Chang, fl., 19 Feb 1955 (as *G. nigrolineata*), *Bunnak 327* (BKF); • ibid., fl. & fr., 11 Mar 1970, *C. F. van Beusekom & T. Santisuk 3156* (AAU, BKF65517, C, E [E00160915], ♂ fl., BKF69931, ♀ fl. & fr.); • ibid., ♀ fl., 11 Mar 1970, *C. F. van Beusekom & T. Santisuk 3165* (AAU, C, E [E00839764], L [L.2409530, L.2409531], P [P05062050]); • Khlong Phlu Waterfall, Ko Chang, fl., 19 Feb 2002 (as *Garcinia* sp.; H. Toyama det. as *G. cochinchinensis*), *T. Wongprasert 022-13* (BKF); • Khlong Makok, Ko Chang National Park, fr., 24 Mar 2001 (as *Garcinia* sp.; H. Toyama det. as *G. delpyana*), *T. Wongprasert 013-04* (BKF132118); • Ko Chang, Route to Wai Cheak Bay, Salak Phet Village, fr., 8 Mar 2023, *H. Balslev et al. 10960* (AAU with photos); • ibid., fr., 17 May 2024, *H. Balslev et al. 11533* (AAU with photos); • Ao Yai, Ko Kut, ♀ fl., 5 Apr 2002, C. *Phengklai et al. 13314* (BKF); • Khlong Chao-Ao Phrao, Ko Kut, fr., 7 Apr 2002 (as *G. merguensis*), *C. Phengklai et al. 13361* (BKF); • Mai Rut Subdistrict, Khlong Yai District, fr., 29 Jun 2012 (as *Garcinia* sp.), *S. Sawangsawat 647* (QBG)]; **Peninsular**: • Chumphon [Ta Ngao, fl., 16 Jan 1927 (as *Garcinia* sp.), *A. F. G. Kerr 11474* (BM, K [K003964719]); • Tha Sae District, fl., 23 Jan 1927 (as *Garcinia* sp.), *A. F. G. Kerr 11624* (BK, BM, K [K003964728]); • Khao Kapo Forest, fr., 20 Jun 1959, *K. Bunchuai 1132* (BKF)]; • Ranong [Kra Buri District, fl., 25 Dec 1928 (as *Garcinia* sp.), *A. F. G. Kerr 16349* (BM, K [K003964720]); • Ko Phayam, Mueang District, fr., 24 Apr 2006 (as *Garcinia* sp.), *T. Wongprasert 064-8* (BKF)]; • Surat Thani [Ko Tao, fl., 12 Apr 1927, *A. F. G. Kerr 12676* (BK, BM, C, K [K003964732]); • Yan Yao Subdistrict, fl., 23 Feb 1930 (as *Garcinia* sp.), *A. F. G. Kerr 18221* (BK, BM, K [K003964727]); • Ko Pha-ngan, fl., 4 Dec 1974, *R. Geesink et al. 7783* (BKF, C, K [K003964700], P [P05061692])]; • Songkhla [Ban Hu Rae, Hat Yai District, ♂ fl., 26 Jun 1986 (as *G. parvifolia*), *G. Junchote 1* (PSU); • Kho Hong Hill, Hat Yai District, ♀ fl., 27 Jul 1986, *J. F. Maxwell 86-516* (AAU, BKF, L [L.2403057], P [P04701445], PSU); • Ban Taling Chan, Chana District, flowers not belonging to *Garcinia*, 20 Apr 1999, *K. Sridith 478* (PSU); • Thamnop Subdistrict, Singhanakhon District, fl., 30 Nov 2000, *K. Sukkapong s.n*. (PSU); • Sathing Mo, Singhanakhon District, fr., 26 Jun 2004, *Wipapan 01* (PSU); • Singhanakhon District, fr., 9 Jul 2011, *M. Wongnawa 2011-01* (PSU)]; • Narathiwat [Hala-Bala Wildlife Sanctuary, Waeng District, fr., 24 Aug 2006, *M. Poopath et al. 96* (BKF, L [L.2403061])].

#### Garcinia oliveri

Taxon classificationPlantaeMalpighialesClusiaceae

Pierre, Fl. Forest. Cochinch. 1(4): 28, t. 64. 1882.

04C57595-43CB-5925-A0E2-8A7C5C2DA624

Figs 5, 6

= Garcinia delpyana Pierre, Fl. Forest. Cochinch. 1(5): 28, t. 65. 1883; Vesque, Epharmosis 2: 22. t. 125, 126. 1889 et in A. DC. & C. DC., Monogr. Phan. 8: 451. 1893; Engl. in Engl. & Prantl, Die Naturlichen Pflanzenfamilien 3(6): 237. 1893; Pit. in Lecomte et al., Fl. Indo-Chine 1(4): 310. 1910; P. H. Hô, Câyco Vietnam 1: 562. fig. 1552. 1991. Type. [Vietnam], Cresit in insula Phú Quốc sinus Siamici [Phú Quốc Island], 23 Jan 1874, *J. B. L. Pierre 3625* (lectotype: designated here, P! [P04701469]; isolectotypes: P! [P04701467, P04701468] and L digital image! [U.1208206]), syn. nov.
= Garcinia curtisii Ridl., Fl. Malay Penins. 1: 175. 1922.
= Garcinia bancana Miq. var. curtisii (Ridl.) Whitmore, Gard. Bull. Singapore 26(2): 276. 1973. Type. Peninsular Malaysia, Penang, from Waterfall, Mar 1892, *C. Curtis 691* (lectotype: first-step designated by Whitmore (1973), without indication of herbarium and barcode; second-step designated here, SING! [SING0069255]; isolectotypes: K! [K000939065], SING! [SING0069254]), syn. nov.

##### Type.

[Vietnam], in sylvis densis ad flumen Bé prope pagum Tri Huyện in Biên Hòa prov. Biên Hòa Gallicae austro Cochinchinae [in dense forests by the Be River near the village of Tri Huyen in Bien Hoa Province of southern Cochinchina], Apr 1873, *J. B. L. Pierre 1373* (lectotype: designated here, P! [P04701853]; isolectotypes: BM digital image! [BM001191361], K! [K000677695], L digital images! [L2417119], P! [P04701837, P04701842, P04701852]).

**Figure 5.**
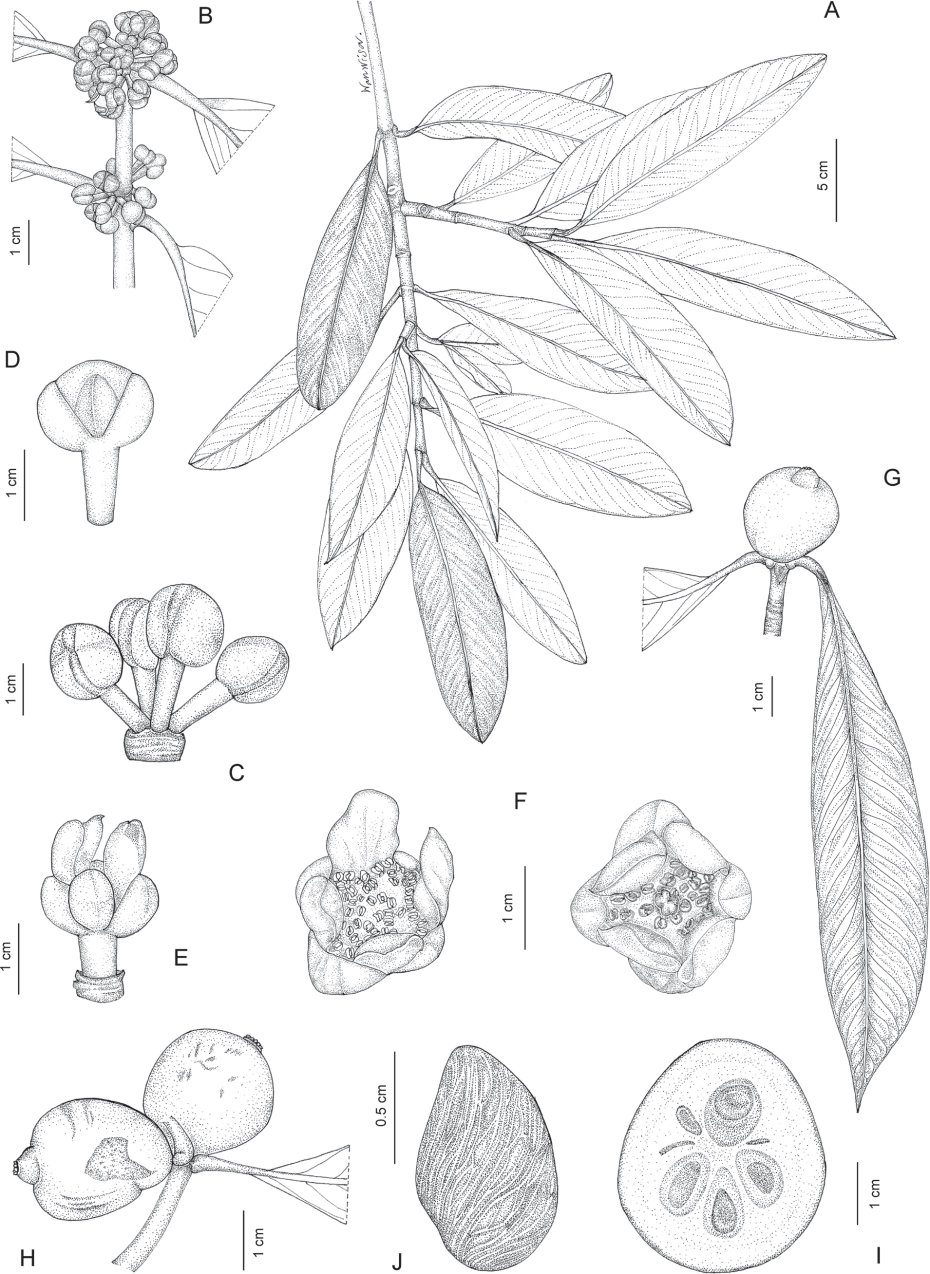
*Garcinia oliveri*. **A**. Branchlets with leaves; **B**. Branchlet with inflorescences bearing staminate flower buds; **C**. Inflorescence with staminate flower buds; **D**. Staminate flower bud in side view; **E**. Staminate flower in side view; **F**. Staminate flowers in top view; **G, H**. Branchlets, leaves, and fruits; **I**. Fruit in transverse section showing seeds with fleshy pulp; **J**. Seed. Photo: Drawn by Wanwisa Bhuchaisri.

**Figure 6.**
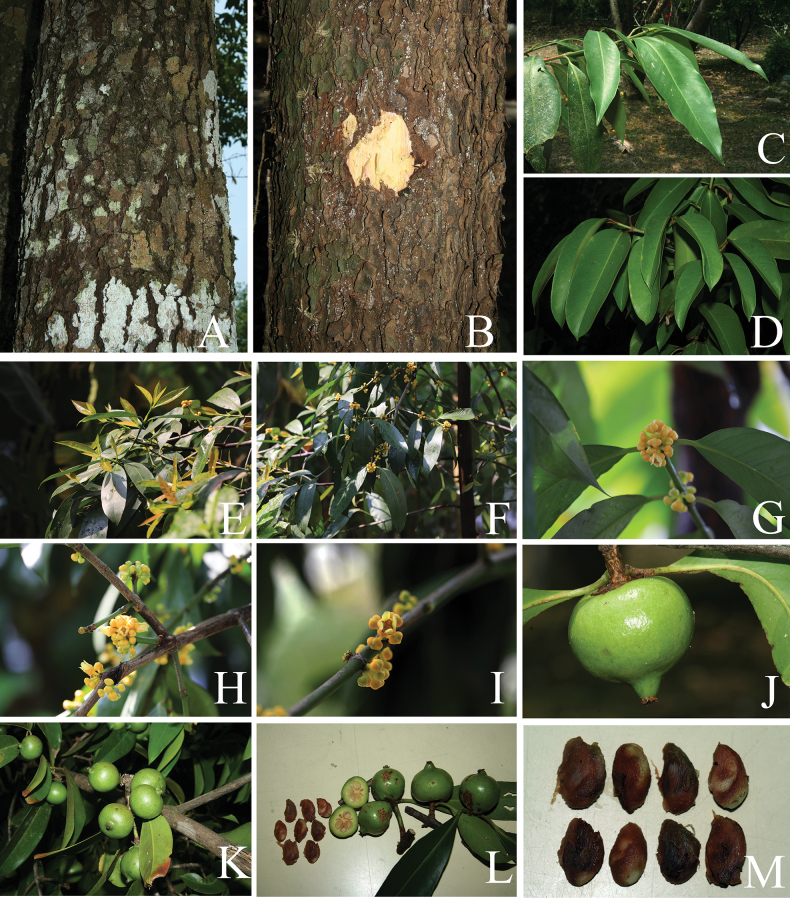
*Garcinia oliveri*. **A**. Bark; **B**. Slashed bark with yellow exudate; **C, D**. Branchlets with leaves; **E**. Branchlets with young leaves; **F**. Branchlets with leaves and inflorescences bearing staminate flower buds; **G–I**. Branchlets with inflorescences bearing staminate flower buds and open staminate flowers; **J, K**. Branchlets with fruits; **L**. Fruits and transverse sections of fruits showing seeds; **M**. Seeds. Photos: Chatchai Ngernsaengsaruay.

##### Description.

Evergreen trees, dioecious, 5–20(–25) m tall, 30–130 cm GBH; exudate pale yellow and sticky; branches decussate, horizontal, or nearly horizontal; branchlets green, 4-angular, glabrous. ***Bark*** brown or dark brown, scaly; inner bark pinkish pale yellow. ***Terminal bud*** concealed between the bases of the uppermost pair of petioles. ***Leaves*** decussate; lamina elliptic or oblong-elliptic, 12–25 × (4–)5.5–9 cm, apex acute or acuminate, base cuneate, margin entire or repand, subcoriaceous, dark green above, paler below, glabrous and glossy on both surfaces, midrib slightly raised (proximal part) and flattened (distal part) above, raised below, secondary veins 10–15 pairs, curving towards the margin and connected in distinct loops and united into an intramarginal vein, flattened above, slightly raised below, with intersecondary veins, veinlets reticulate, visible above, interrupted long wavy lines present, of differing lengths, running across the secondary veins to the apex; petiole green, sometimes with the uppermost pair red or greenish red, turning green with age, 1.5–2.5 cm long, 0.3–1 cm diam., slightly raised above, glabrous, with a basal appendage clasping the branchlet; fresh leaves brittle when crushed; young leaves red or brownish red, turning pale green, glossy. ***Inflorescences*** terminal or borne on short, leafless lateral branchlets, in fascicles of 3–9-flowered cymes (staminate inflorescence usually bearing more flowers than pistillate ones), or sometimes a solitary flower (in pistillates); bracts early caducous. ***Flowers*** unisexual, 4-merous; pedicel greenish yellow to yellow, glabrous; bracteoles early caducous; sepals and petals decussate, glabrous, slightly thick, and fleshy; sepals yellow to orangish yellow, concave; petals pale yellow to yellow, not concave. ***Flower buds*** pale green, becoming greenish yellow, yellow to orangish yellow before anthesis, subglobose or globose, 3–6 mm diam. ***Staminate flowers*** 1–1.2 cm diam.; pedicel 4–7 mm long, 1.5–3.5 mm diam.; sepals 4, broadly elliptic or suborbicular, 5–8 × 4.5–8 mm, the outer pair slightly larger than the inner pair, apex rounded; petals 4, broadly oblong, broadly elliptic, or elliptic, 0.9–1.3 × 0.7–1.3 cm, subequal, apex rounded; stamens numerous, united into a single central 4-sided or weakly 4-lobed bundle surrounding a pistillode or without a pistillode, bundle 5–7 mm diam; filaments very short; anthers small, 4-thecous, longitudinally dehiscent; pistillode fungiform, 2–3.5 mm long. ***Pistillate flowers*** 0.8–1.3 cm diam.; pedicel 3–4.5 mm long, 2.5–4 mm diam.; sepals and petals same as in staminate flowers; staminodes 5–11, united into 4 bundles, surrounding the ovary; pistil fungiform, 4–6 mm long; ovary pale green, subglobose or globose, 5–7 × 5–7.5 mm; stigma pale yellow, convex, radiate, shallowly 6–10-lobed, 4–5.5 mm diam., papillate. ***Fruits*** berries, green, turning yellow or orangish yellow when ripe, glabrous, cut fruits with a sticky yellow exudate, variable in shape, subglobose, globose, broadly ellipsoid, or sometimes broadly obovoid, 3.5–5 × 3–4.5 cm, sometimes oblique, asymmetrical, unlobed, sometimes weakly 6–10-lobed, with a short, thick beak at the apex, pericarp fleshy, 2–4 mm thick; persistent stigma dark brown or brown, 5–6 mm diam., weakly 6–10-lobed, papillate; persistent sepals slightly larger than in flowering material; fruiting stalk short and thick, 3–6.5 mm long, 3–6 mm diam. ***Seeds*** 6–10, sometimes aborted (1–5), dark brown mottled with paler irregular lines, semi-ellipsoid, 1.4–2.7 × 0.9–1.3 cm, rounded at both ends, with a yellow to orangish yellow fleshy pulp.

##### Distribution.

Vietnam, Laos, Cambodia, Thailand, and Peninsular Malaysia (Penang, Kelantan, Kepong) (Fig. 4B).

##### Distribution in Thailand.

**North-Eastern**: Loei, Buengkan; **Eastern**: Nakhon Ratchasima, Ubon Ratchathani; **Central**: Saraburi, Nakhon Nayok; **South-Eastern**: Chanthaburi, Trat; **Peninsular**: Surat Thani, Phangnga, Phuket, Nakhon Si Thammarat, Phatthalung, Trang, Satun, Songkhla, Narathiwat (Fig. 4B).

##### Habitat and ecology.

It is found in dry evergreen forests, tropical lowland evergreen rain forests, peat swamp forests, and secondary forests, occasionally occurring along streams at elevations ranging from near sea level to 800 m a.m.s.l.

##### Phenology.

Flowering and fruiting occur more than once, nearly throughout the year; flowering peaks from December to April, and fruiting peaks from April to August.

##### Conservation status.

*Garcinia oliveri* is widely distributed from Indo-China to Peninsular Malaysia. It is known from many localities and has a large EOO of 1,086,628.84 km^2^ and an AOO of 196 km^2^. In Thailand, this species has an EOO of 798,570.92 km^2^ and an AOO of 128 km^2^. Because of its wide distribution, the number of localities, and the absence of any apparent imminent threats to the plants or their habitats, we assess its conservation status here as LC, in agreement with de Kok (2024a).

##### Etymology.

The specific epithet of *Garcinia oliveri* honors the British botanist Daniel Oliver (1830–1916), who served as assistant at the Kew Herbarium (1858–1864), later as its keeper (1860–1890), and was also a professor of botany at University College, London (1861–1888) (Stafleu and Cowan 1981). He contributed significantly to tropical botany.

##### Vernacular names.

**Cha muang bai yai** (ชะมวงใบใหญ่) (suggested here); Cha muang chang (ชะมวงช้าง) (Trat, from the specimen *K. Bunchuai 13*); Muang (มวง) (Satun, from the specimen *A. F. G. Kerr 14200*); Som mong (ส้มโมง) (Saraburi, from the specimen *A. F. G. Kerr 10005*); U mong (อุโมง) (Trat, from the specimen *Put 563*).

##### Uses.

It can be utilized in a similar way to *Garcinia cowa* var. *cowa*.

##### Lectotypifications.

*Garcinia oliveri* was described by Pierre (1882) based on seven gatherings: *Herb. Pierre 2*, *7720*, *1373*, *3624*, *3626*, and *3628*, collected from various parts of Lower Cochinchina and Cambodia (“Habite toutes les parties de la Basse-Cochinchine et du Cambodge”), and *Harmand 190*, collected from Southern Laos (“le Laos méridional”). Pierre did not indicate a type, nor did he indicate the herbaria in which the specimens were deposited. Following Art. 9.6 of the ICN (Turland et al. 2018), all these specimens are therefore considered syntypes. However, detailed examination revealed that specimens *J. B. L. Pierre 2*, *1373*, and *3628* differ in both their localities and dates of collection, but we did not find specimen *J. B. L. Pierre 3624*.

The specimen *J. B. L. Pierre 2* can be separated into four distinct groups:

Group A: Collected from the Deon Ba Mountains in Tây Ninh Province, southern Cochinchina (“Crescit montibus Deon Ba, prov. Tây Ninh austro Cochinchinae”) on 15 April 1866. Specimen housed at: P [P04701526].

Group B: Collected from the Dinh Mountains in Baria Province, southern Cochinchina (“Crescit in montibus Dinh in prov. Baria Gallicae austro Cochinchinae”) in June 1866. Specimens housed at: P [P04701527, P04701529, P0470153].

Group C: Collected from the Dinh Mountains (Mu xoai) in Baria Province, southern Cochinchina (“Oritur in montibus Dinh (Mú xoài) in prov. Baria Gallicae austro Cochinchinae”) in September 1866. Specimen housed at: P [P04701528].

Group D: Collected from the Dinh Mountains (Mu xoai) in Baria Province, southern Cochinchina (“Oritur in montibus Dinh (Mú xoài) in prov. Baria Gallicae austro Cochinchinae”) on 14 March 1867. Specimens housed at: K [K000677694], P [P04701862, P04701865, P04701866], and U [U1199443].

Upon examination, the specimens cited as number “*7720*” in the original description correspond in all respects to the specimen labelled “*772*,” and no distinct gathering corresponding to “*7720*” was located. We therefore interpret the citation of “*Herb. Pierre 7720*” in the protologue as a transcription error and correct it to “*J. B. L. Pierre 772*.” The specimen *J. B. L. Pierre 772*, collected from the Schnal Mountains (“in montibus Schnal”), Cambodia, in May 1870, is housed at: K [without barcode].

The specimen *J. B. L. Pierre 1373* can be separated into three distinct groups:

Group A: Collected from the Dinh Mountains (Mu xoai) in Baria Province, southern Cochinchina (“Oritur in montibus Dinh (Mú xoài) in prov. Baria Gallicae austro Cochinchinae”) on 14 March 1867. Specimens housed at: BM [BM001191363], K [without barcode], L [L2417118], and P [P04701849].

Group B: Collected in dense forests by the Be River near the village of Tri Huyen in Bien Hoa Province of southern Cochinchina (“in sylvis densis ad flumen Bé prope pagum Tri Huyện in Biên Hòa prov. Biên Hòa Gallicae austro Cochinchinae”) in April 1873. Specimens housed at: BM [BM001191361], K [K000677695], L [L2417119], and P [P04701837, P04701842, P04701852, P04701853].

Group C: Collected near the village of Giang Dong on Phu Quoc Island (“ad pagum Giang Đông in insula Phú Quốc sinus Siamici”) in December 1874. Specimens housed at: BM [BM001191364], K [without barcode], L [L0700336], and P [P04701850].

The specimen *J. B. L. Pierre 3626* collected from the village of Giang Dong on Phu Quoc Island (“crescit ad pagum Giang Đông in insula Phú Quốc sinus Siamici”) in December 1874, is housed at: BM [BM001191362], CAL [CAL0000005848], P [P04701844, P04701845, P04701847], and U [U1199444].

The specimen *J. B. L. Pierre 3628* can be separated into two distinct groups:

Group A: Collected from the Dinh Mountains in Baria Province, southern Cochinchina (“Crescit in montibus Dinh in provincia Baria austro Cochinchinae”) in June 1866. Specimen housed at: P [P04701851].

Group B: Collected from the Dinh Mountains near Baria, southern Cochinchina (“Crescit in montibus Dinh juxta Baria Gallicae austro Cochinchinae”) April 1867. Specimen housed at: P [P04701846].

The specimen *F. J. Harmand 190* collected from Selamphao, Sè-Moun Basin, Southern Laos [“Selamphao, Bassin du Sè-Moun (Laos méridional)”] in Jan 1876 is housed at: P [P04701858, P04701859].

The specimens cited under *J. B. L. Pierre 1373* represent three distinct gatherings differing in locality and collection date. When multiple gatherings are involved, the choice of a lectotype must be justified by reference to the morphological characters given in the protologue. Among the syntypes, the specimen of Group B at P [P04701853] is the most complete and best preserved and shows the closest correspondence to the morphology described in the protologue. We therefore designate *J. B. L. Pierre 1373* (Group B) at P [P04701853] as the lectotype, following Art. 9.3 of the ICN (Turland et al. 2018). The remaining duplicates of the same gathering at BM [BM001191361], K [K000677695], L [L2417119], and P [P04701837, P04701842, P04701852] are designated as isolectotypes, following Art. 9.12 of the ICN (Turland et al. 2018)

*Garcinia delpyana* was described by Pierre (1883) based on three gatherings: *Herb. Pierre 2002*, *3624*, and *3635*, collected along the coast of Pile Phu-Quoc and in Kampot Province (“Cette espèce est commune sur le littoral de Pile Phu-Quoc et dans la province de Kamput”). Pierre did not indicate a type, nor did he specify the herbaria in which the specimens were deposited. Following Art. 9.6 of the ICN (Turland et al. 2018), all of these specimens are therefore considered syntypes.

The specimen *J. B. L. Pierre 2002*, collected from Phu Quoc (“Phú Quốc sinus Siamici”) in March 1877, is housed at: P [P04701474].

The specimen *J. B. L. Pierre 3624*, collected from the Cám Chay Mountains in Kampot Province (“Cresit in montibus Cám Chay in prov. Kamput”) in April 1874, is housed at: P [P04701472, P04701473].

Upon examination, the specimens cited as number “*3635*” in the original description correspond in all respects to the specimen labelled “*3625*,” and no distinct gathering corresponding to “*3635*” was located. We therefore interpret the citation of “*Herb. Pierre 3635*” in the protologue as a transcription error and correct it to “*J. B. L. Pierre 3625*.” Specimens under the collection number *3625* differ in their dates of collection and can be separated into two distinct groups:

Group A: Collected from Giang Dong on Phú Quốc Island (“ad Giang Dong in insula Phú Quốc sinus Siamici”) on 18 January 1874. Specimens housed at: P [P04701470, P04701471].

Group B: Collected from Phú Quốc Island (“Cresit in insula Phú Quốc sinus Siamici”) on 23 Jan 1874. Specimens housed at: P [P04701467, P04701468, P04701469] and L [U.1208206].

We hereby designate the specimen *J. B. L. Pierre 3625* from Group B at P [P04701469] as the lectotype, as it is the most complete and best-preserved specimen among the syntypes, in accordance with Art. 9.3 of the ICN (Turland et al. 2018). The remaining specimens from the same gathering at P [P04701467, P04701468] and L [U.1208206] are designated as isolectotypes, following Art. 9.12 of the ICN (Turland et al. 2018).

In the original publication of *Garcinia curtisii* Ridl. by Ridley (1922), two gatherings were mentioned: *C. Curtis 691* and *240*, collected from Penang, near the Waterfall and at Teluk Bahang (“Telok Bahang”). The name *G. curtisii* was lectotypified in a first-step by Whitmore (1973), based on the specimen *C. Curtis 691*, but he did not indicate the herbarium where the specimen is housed. However, upon examination, we found that specimens under this collection number differ in both locality and collection date and can be separated into three distinct groups:

Group A: *C. Curtis 691* from Teluk Bahang, Penang, collected in March 1886. Specimen at: K [K000939066].

Group B: *C. Curtis 691* from Waterfall near Upper Bridge, Penang, collected in March 1890. Specimen at: SING [SING0069252].

Group C: *C. Curtis 691* from Waterfall, Penang, collected in March 1892. Specimens at: K [K000939065] and SING [SING0069254, SING0069255].

Therefore, we designate the specimen *C. Curtis 691* from Group C at SING [SING0069255] as the second-step lectotype, with two sheets of isolectotypes at K [K000939065] and SING [SING0069254].

##### Notes.

According to previous studies (e.g., Pierre 1882, 1883; Vesque 1889, 1893; Pitard 1910; Gagnepain 1943) and based on the specimens we examined, *Garcinia delpyana* is not morphologically distinguishable from *G. oliveri* and is therefore treated here as a new synonym of the latter.

Whitmore (1973) reduced *Garcinia curtisii* to a variety under *G. bancana*, as *G. bancana* Miq. var. curtisii (Ridl.) Whitmore. However, based on previous studies (e.g., Whitmore 1973; Ridley 1922) and our examination of specimens, *G. bancana* var. curtisii is not morphologically distinguishable from *G. oliveri* and is therefore treated here as a new synonym of that species.

*Garcinia oliveri* is similar to *G. cowa* var. *cowa* and is distinguished by its leaves elliptic or oblong-elliptic and larger, 12–25 × (4–)5.5–9 cm; secondary veins 10–15 pairs; and fruits subglobose, globose, broadly ellipsoid, or sometimes broadly obovoid, 3.5–5 × 3–4.5 cm, unlobed or sometimes weakly lobed, with a short, thick beak at the apex. In contrast, *G. cowa* var. *cowa* has leaves narrowly elliptic, elliptic, lanceolate, lanceolate-ovate, or ovate and smaller 5–15 × 3–6 cm; secondary veins 6–10 pairs; and fruits subglobose, globose, depressed globose, or broadly ellipsoid, 3–7 × 2.5–5 cm, shallowly or weakly lobed, without or with a short, thick beak at the apex.

The specimen *T. Wongprasert 013-04*, collected from Khlong Makok, Ko Chang National Park, Trat Province, on 24 March 2001, is represented at BKF by two sheets under the same collector’s number: BKF132118, identified as *Garcinia oliveri*, and BKF132119, identified as *G. merguensis* Wight.

The *specimen C. Phengklai et al. 14603*, collected from Ko Kut, Trat Province, on 6 April 2002, is represented at BKF by two sheets under the same collector’s number: BKF142164, identified as *Garcinia oliveri*, and BKF143190, identified as *G. lanceifolia* Roxb. (syn. *G. gracilis* Pierre).

##### Additional specimens examined.

**Thailand. North-Eastern**: • Loei [Huai Baeng Forest Protection Station, Phu Luang Wildlife Sanctuary, Wang Saphung District, fr., 11 Jun 2004 (as *Garcinia* sp.), *T. Wongprasert et al. 046-14* (BKF); • ibid., fl., 10 Mar 2009 (H. Toyama det. as *G. delpyana*), *T. Wongprasert 093-24* (BKF); • Buengkan [Wat Chotirot Thammakhon, Buengkan Subdistrict, Buengkan District, fr., 14 Jun 2004 (as *Garcinia* sp.), *T. Wongprasert et al. 046-84* (BKF)]; **Eastern**: • Nakhon Ratchasima [Wang Nam Khiao District, fr., 22 Jan 1968 (as *Garcinia* sp.), *Damrongsak 524* (BKF112733); • Pak Thong Chai District, fl., 28 Feb (year unspecified) (as *Garcinia* sp.; H. Toyama det. as *G. oliveri*), *CH 345* (BKF112735)]; • Ubon Ratchathani [Chong Mek, fl., 16 Feb 1967 (as *Garcinia* sp.), *S. Phusomsaeng 16* (BKF, K); • Dong Fa Huan Botanical Garden, Mueang Ubon Ratchathani District, fr., 1 May 2002 (H. Toyama det. as *G. delpyana*), *R. Pooma et al. 3318* (BKF, QBG, SING [SING0095615]); • Phu Sa Yak, Buntharik District, fl., 22 Jan 2005 (H. Toyama det. as *G. oliveri*), *P. Puudjaa 1366* (BKF); • Phu Chong Na Yoi National Park, Na Chaluai District, fr., 15 Jun 2005 (as *Garcinia* sp.), *K. Wangwasit 050615-49* (QBG)]; **Central**: • Saraburi [Muak Lek District, ♂ fl., 19 Jan 1925 (H. Toyama det. as *G. delpyana*), *A. F. G. Kerr 10005* (BM, K [K003964607])]; • Nakhon Nayok [Sarika Waterfall, fr., 14 Aug 1968 (H. Toyama det. as *G. cf. delpyana*), *K. Larsen et al. 3413* (AAU, BKF, C, E [E00839767], K, P); • Wang Ta Khrai, Mueang Nakhon Nayok District, fr., 28 Mar 2000 (H. Toyama det. as *G. delpyana*), *T. Wongprasert s.n*. (BKF127948); • Khao Yai National Park, fr., 9 Jun 2000 (H. Toyama det. as *G. delpyana*), *C. Niyomdham 6216* (BKF); • Khao Yai National Park, Mo Sing To area, ♂ fl., 13 Mar 2002 (as *G. benthamii*), *J. F. Maxwell 02-92* (BKF, CMUB); • ibid., fr., 10 Aug 2002 (as *G. benthamii*), *A. Boonkongchart & C. Chongko 140* (BKF, CMUB); • ibid., sterile, 7 Jul 2003 (as *G. benthamii*), *J. F. Maxwell & A. Boonkongchart 4* (CMUB)]; **South-Eastern**: • Chanthaburi [Laem Sing District, fl., 23 Nov 1924 (as *Garcinia* sp.), *A. F. G. Kerr 9372* (BK)]; • Trat [Khao Saming District, fl., 26 Jan 1927 (as *Garcinia* sp.), *Put 563* (BK, K); • Laem Ngop District, fr., 24 Feb 1954 (as *Garcinia* sp.), *K. Bunchuai 13* (BKF); • Khlong Son, Ko Chang, fr., 28 Feb 1900 (as *G. nigrolineata*), *J. Schmidt 647a* (C); • Ko Chang, fr., 11 Mar 1970, *C. F. van Beusekom & T. Santisuk 3160* (AAU, C, E [E00839768], K, L [L.2409523], P); • Khlong Ma Yom, Ko Chang, fr., 9 May 1974 (as *G. speciosa* and *Garcinia* sp.), *J. F. Maxwell 74-431* (AAU, BK); • Khlong Phlu Waterfall, Ko Chang, fr., 22 Mar 2001 (as *Garcinia* sp.; H. Toyama det. as *G. delpyana*), *K. Chayamarit et al. 2781* (BKF); • ibid., fr., 23 Mar 2001 (as *Garcinia* sp.; H. Toyama det. as *G. delpyana*), *K. Chayamarit et al. 2819* (BKF); • Khlong Makok, Ko Chang National Park, fr., 24 Mar 2001 (as *Garcinia* sp.; H. Toyama det. as *G. delpyana*), *K. Chayamarit et al. 2925* (BKF, SING [SING0095614]); • Ko Chang, ♂ fl., 7 Mar 2003 (as *G. cowa*; H. Toyama det. as *G. delpyana*), *C. Phengklai et al. 14198* (BKF); • Ko Mak, fl., 18 Nov 1966 [as *Garcinia* sp.; H. Toyama det. as *G. delpyana*; C. Ngernsaengsaruay det. as *G. nigrolineata* (C)], *B. Hansen & T. Smitinand 12369* (BKF, C, K); • Ko Kut, fl., 22 Oct 2000 (as *G. cowa*; H. Toyama det. as *G. delpyana*), *C. Phengklai et al. 13144* (BKF); • Ko Kut, fr., 6 Apr 2002 (as *G. cf. atroviridis*; H. Toyama det. as *G. delpyana*), *C. Phengklai et al. 14603* (BKF142164); • Khlong Chao-Ao Phrao, Ko Kut, fr., 7 Apr 2002 (as *G. cf. cowa*; H. Toyama det. as *G. delpyana*), *C. Phengklai et al. 13452* (BKF); • Ao Salat, Ko Kut, fr., 9 Apr 2002 (as *G. cf. lanessanii*), *C. Phengklai et al. 13604* (BKF); • Ko Kut, fl., 2 Dec 2006 (as *Garcinia* sp.), *C. Phengklai et al. 15432* (BKF); **Peninsular**: • Surat Thani [Big Tree Nature Trail, Khlong Phanom National Park, Phanom District, fl., 23 Oct 2004 (as *Garcinia* sp.), *S. Gardner & P. Sidisunthorn ST1094* (K)]; • Phangnga [Khao Pok Hill, Krasom Subdistrict, Takua Pa District, fl., 2 Dec 1918 (as *Garcinia* sp., *G. nigrolineata*), *Haniff 3920* (K, SING); • Khao Lak-Lam Lu National Park, Takua Pa District, fr., 11 Jun 2004 (as *Garcinia* sp.), *S. Gardner & P. Sidisunthorn ST0716* (K); • Ko Yao Yai, Ko Yao District, fr., 30 Apr 2007 (H. Toyama det. as *G. cf. delpyana*), *T. Wongprasert 074-13* (BKF)]; • Phuket [Ton Sai Waterfall to Pa Khlok, ♂ fl., 7 Jul 1979 (as *G. nigrolineata*, H. Toyama det. as G. delpyana), *C. Niyomdham et al. 229* (AAU, BKF, C)]; • Nakhon Si Thammarat [Ka Piat Subdistrict, ♂ fl., 15 Mar 1957 (H. Toyama det. as *G. delpyana*), *Snan 1003* (BKF); • Tha Phae Waterfall, Chang Klang District, fr., 11 Jul 2000 (H. Toyama det. as *G. delpyana*), *V. Chamchumroon VC841* (BKF)]; • Phatthalung [Tamot Waterfall, fr., 9 Aug 1986 (as *G. nigrolineata*, H. Toyama det. as *G. delpyana*), *J. F. Maxwell 86-549* (BKF, L [L2416709, L2416708], P [P04701556], PSU); • Khao Pu-Khao Ya National Park, Si Banphot District, fr., 15 Jul 2000 (as *G. nigrolineata*), *D. J. Middleton et al. 47*2 (AAU, K)]; • Trang [Khao Chong, fl., 18 Nov 1965 (as *Garcinia* sp.; H. Toyama det. as *G. delpyana*), *C. Boonnab 168* (BKF)]; • ibid., fl., Dec 1974 (H. Toyama det. as *G. delpyana*), *B. Nimanong et al. 1624* (BKF, C, K [K003964608], L [L.2409513], P); • ibid., fr., 12 Aug 1975 (as *Garcinia* sp.), *J. F. Maxwell 75-804* (AAU, BK); • ibid., ♂ fl., 1 Feb 1985 (as *G. nigrolineata*, H. Toyama det. as *G. delpyana*), *J. F. Maxwell 85-129* (BKF, PSU); • ibid., fl., Feb 2001 (H. Toyama det. as *G. delpyana*), *A. Sinbumroong & S. Davies AS209* (BKF); • ibid., fr., Feb 2001 (as *Garcinia* sp.), *A. Sinbumroong & S. Davies AS226* (BKF)]; • Satun [Tarutao National Park, fl., 20 Jan 1928 (as *Garcinia* sp., *G. cf. cornea*), *A. F. G. Kerr 14200* (BK, K); • Trail between Talo Wao and Talo Udang, Tarutao National Park, fl., 9 Feb 2005 (as *Garcinia* sp.), *P. Sidisunthorn & P. Tippayasri ST1472* (K); • Ao Son, Tarutao National Park, La-ngu District, fl., 10 Feb 2005 (as *Garcinia* sp.), *P. Sidisunthorn & P. Tippayasri ST1487* (K); • Tarutao National Park, fr., 8 Apr 2008 (as *G. cowa*; H. Toyama det. as *G. delpyana*), *C. Phengklai et al. 15761* (BKF)]; • Songkhla [Hat Yai, Kho Hong Hill, fr., 17 Sep 1981 (as *G. nigrolineata*), *P. Sirirugsa 462* (PSU); PSU campus, base of Kho Hong Hill, ♂ fl., 21 Mar 1985 (as *G. nigrolineata*), *P. Sirirugsa 1002* (AAU, BKF, E [E00839769], L [L2416710, L2416711], PSU); • Khlong Rhang Hill, Na Mom District, ♂ fl., 22 Mar 1985 (as *G. nigrolineata*), *J. F. Maxwell 85-322* (AAU, BKF, E [E00160906], L [L.2416684, L.2416685]; PSU); • Kho Hong Hill, Hat Yai District, fr., 27 July 1985 (as *G. nigrolineata*, H. Toyama det. as *G. delpyana*), *J. F. Maxwell 85-757* (AAU, BKF, L [L2416686], PSU, P [P04701605]); • Ton Nga Chang Wildlife Sanctuary, Hat Yai District, fl., 20 Mar 2004 (as *Garcinia* sp.), *S. Gardner & P. Sidisunthorn ST0266* (K); • PSU Campus, Kho Hong Hill, Hat Yai District, sterile, 24 Mar 2008 (as *G. nigrolineata*), *N. Boonnak NW-005* (PSU)]; • Narathiwat [Bacho, fl., 15 Jul 1923 (as *Garcinia* sp.), *A. F. G. Kerr 7209* (K); • ibid., fl., 15 Jul 1923 (as *Garcinia* sp.), *A. F. G. Kerr 7211* (BK, K); • ibid., fl., 18 Apr 1961 (as *Garcinia* sp.), *B. Sangkhachand 67* (K); • Ban Yuan Yang, Su-ngai Padi District, ♀ fl., 7 Jun 1987 (as *G. bancana* var. curtisii), *J. F. Maxwell 87-531* (PSU); • ibid., ♀ fl., 7 Jun 1987 (as *G. bancana* var. curtisii), *J. F. Maxwell 87-532* (BKF, L [L2409055, L2409056])].

#### Garcinia schomburgkiana

Taxon classificationPlantaeMalpighialesClusiaceae

Pierre, Fl. Forest. Cochinch. 1(5): 28, t. 82A. 1883.

01727129-A8E2-5BDE-B55D-6F8FE3F1F5FA

Figs 7, 8, 9

##### Type.

[Thailand], Siam, Espèce commune dans le delta du Menam [a common species in the Chao Phraya River, 1859, *R. Schomburgk s.n*. (in the original description may be a transcription error as “*Schomburgk 1151*”) (lectotype: designated here, P! [P04701350]; isolectotypes: K! [K000677697, K000677698]).

**Figure 7.**
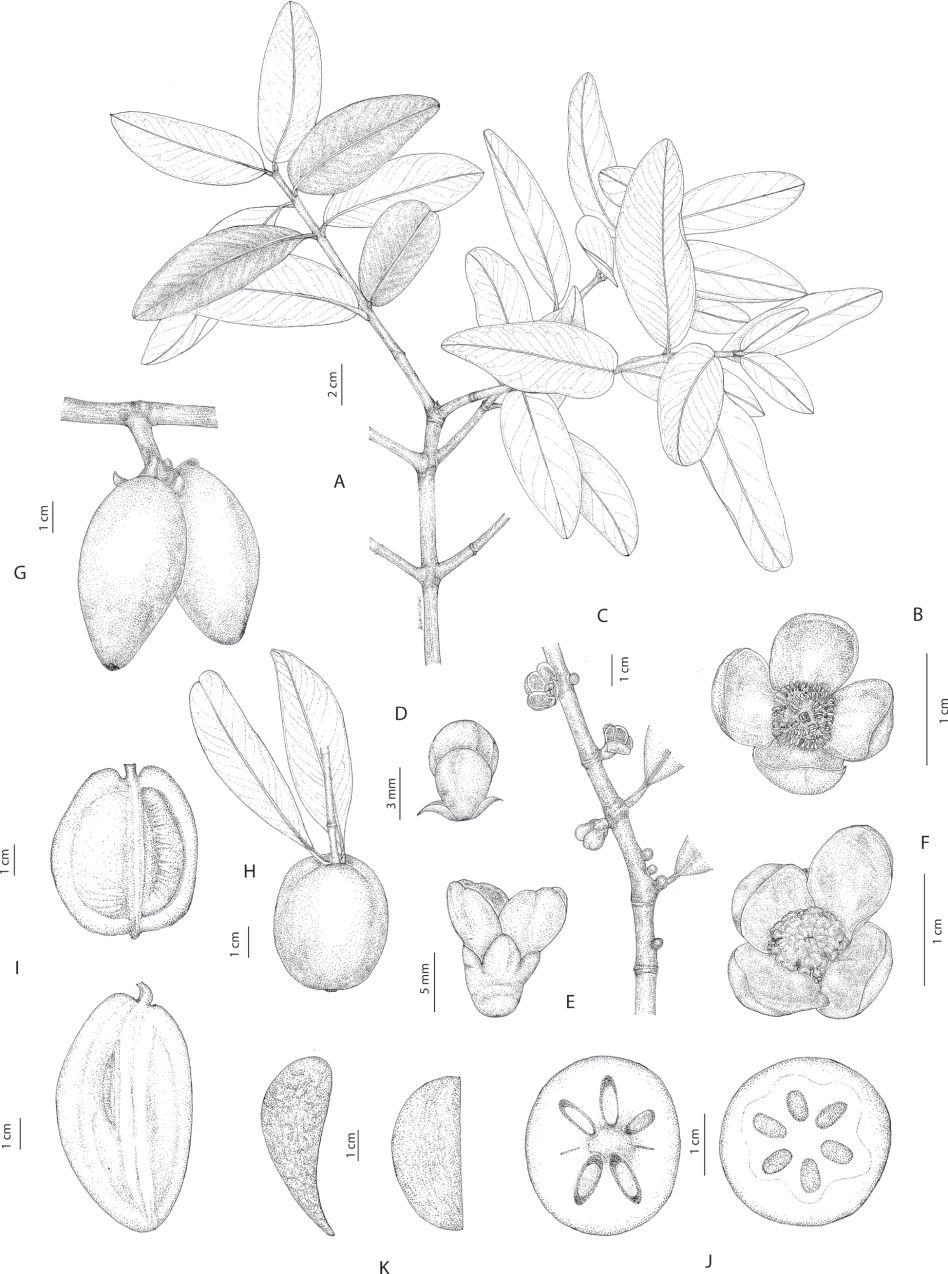
*Garcinia schomburgkiana*. **A**. Branchlets with leaves; **B**. Staminate flower in top view; **C**. Branchlet with pistillate flower buds and open pistillate flowers; **D**. Pistillate flower bud; **E**. Pistillate flower in side view; **F**. Pistillate flower in top view; **G, H**. Fruits; **I**. Fruits in longitudinal section; **J**. Fruits in transverse section showing seeds with fleshy pulp; **K**. Seeds. Photo: Drawn by Wanwisa Bhuchaisri.

**Figure 8.**
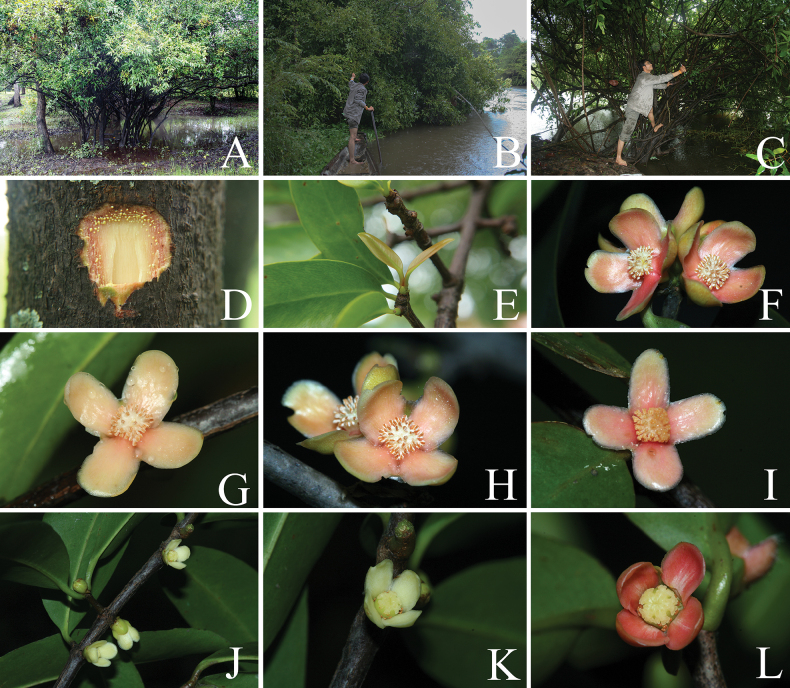
*Garcinia schomburgkiana*. **A–C**. Habit and habitats; **D**. Slashed bark with yellow exudate; **E**. Branchlet with young leaves; **F–I**. Branchlets with inflorescences bearing open staminate flowers; **J–L**. Branchlets with leaves and inflorescences bearing open pistillate flowers. Photos: Chatchai Ngernsaengsaruay (**A–D, F–L**); Pichet Chanton (**E**).

**Figure 9.**
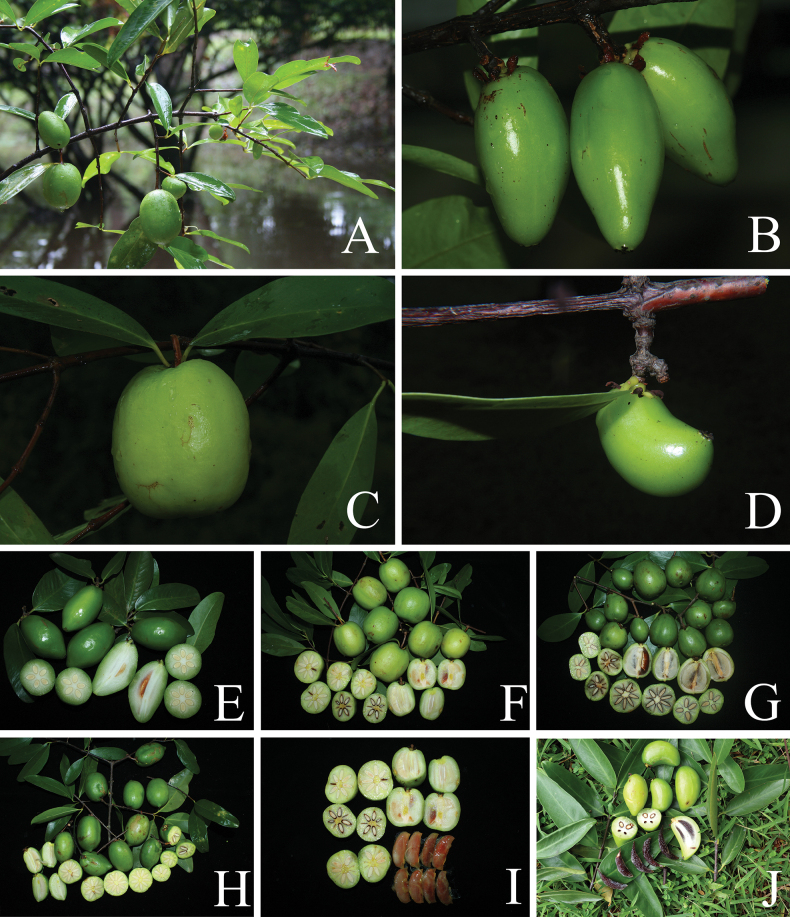
*Garcinia schomburgkiana*. **A**. Branchlets with leaves and fruits; **B–D**. Branchlets with fruits; **E–J**. Fruits, transverse and longitudinal sections of fruits showing yellow exudate and seeds. Photos: Chatchai Ngernsaengsaruay (**A–C, E–I**); Pichet Chanton (**D, J**).

##### Description.

Small evergreen trees, monoecious or dioecious, 3–7 m tall, 30–80 cm GBH, much-branched, with spreading branches; exudate pale yellow to yellow and sticky; branchlets decussate; young branchlets green, 4-angular, glabrous. ***Bark*** dark brown or dark grayish brown, smooth; inner bark pinkish red. ***Terminal bud*** concealed between the bases of the uppermost pair of petioles. ***Leaves*** decussate; lamina elliptic, narrowly elliptic or oblong-elliptic, 5–14 × 2.5–4 cm, apex acute or obtuse, base cuneate, margin entire, subcoriaceous, dark green above, paler below, glabrous and glossy on both surfaces, midrib slightly raised (proximal part) and flattened (distal part) above, raised below, secondary veins 8–11 each side, curving towards the margin and connected in distinct loops and united into an intramarginal vein, flattened above, slightly raised below, with intersecondary veins, veinlets reticulate, visible above, interrupted long wavy lines present, of differing lengths, running across the secondary veins to the apex; petiole green, 0.5–1.2 cm long, 1–3 mm diam., slightly raised above, glabrous, with a basal appendage clasping the branchlet; fresh leaves brittle when crushed; young leaves brownish red, turning pale green, glossy. ***Inflorescences*** terminal or borne on short, leafless lateral branchlets, usually fascicled, 2–3(–4)-flowered cymes in staminates or a solitary flower in both sexes; bracts 2, green, broadly ovate, 2–3.5 × 2–3 mm, apex acute. ***Flowers*** unisexual, 4-merous; pedicel pale green, 1.5–3.5 mm long, 1.5–4 mm diam., thick, glabrous; bracteoles same as bracts; sepals and petals decussate, glabrous, slightly thick and fleshy; sepals pale green, concave; petals variable in color, pale yellow, yellow, pinkish yellow, yellowish pink, pink, pinkish red or red, slightly concave to concave or not concave. ***Flower buds*** subglobose, 3–4 mm diam.; young flower buds pale green; mature flower buds similar in color to open flowers. ***Staminate flowers*** with spreading petals, 1.7–2.7 cm diam.; sepals 4, broadly elliptic, elliptic or broadly ovate, 3–4.5 × 4–6 mm, the outer pair slightly larger than the inner pair, apex rounded; petals 4, variable in shape, broadly elliptic, elliptic, broadly oblong, obovate, oblanceolate or oblanceolate-obovate, 0.6–1.4 × (0.2–)0.4–0.8 cm, subequal, apex rounded; stamens numerous, united into a single central 4-sided or weakly 4-lobed bundle, bundle 4–6 mm diam; filaments very short; anthers small, 4-thecous, longitudinally dehiscent; pistillode absent. ***Pistillate flowers*** with slightly spreading petals, 4–6 mm diam.; sepals and petals same as in staminate flowers; staminodes 12–15, united into 4 bundles, surrounding the ovary; pistil fungiform, 4–6 mm long; ovary pale green, subglobose or globose, 3–5 × 3.5–5 mm; stigma pale yellow, convex, radiate, shallowly 5–9-lobed, 5–7 mm diam., papillate. ***Fruits*** berries, green or pale green, glabrous and glossy, cut fruits with a sticky pale yellow to yellow exudate, variable in shape, ovoid, broadly ovoid, ellipsoid, broadly ellipsoid, sometimes obovoid, broadly obovoid, or subglobose, 4–9 × 2.5–5 cm, sometimes oblique, asymmetrical, unlobed, with a short, thick beak at the apex, pericarp fleshy, 5–7 mm thick; persistent stigma dark brown or blackish brown, 0.7–1.1 cm diam., weakly 5–9-lobed, papillate; persistent sepals slightly larger than in flowering material; fruiting stalk short and thick, 5.5–7 mm long, 4–6 mm diam. ***Seeds*** 5–9, sometimes aborted (1–4), dark brown or brown, crescent-shaped and acuminate at both ends or semi-ellipsoid and rounded at both ends, 3–8 × 0.7–2 cm, with a white fleshy pulp.

##### Distribution.

Vietnam (reported by Hô 1991), Cambodia, Thailand (Fig. 4C).

##### Distribution in Thailand.

**Northern**: Chiang Mai, Lampang (expected to be cultivated in the northern region); **North-Eastern**: Bueng Kan, Nakhon Phanom, Maha Sarakham; **Eastern**: Roi Et, Si Sa Ket, Ubon Ratchathani; **Central**: Ang Thong, Pathum Thani, Bangkok; **Peninsular**: Surat Thani, Songkhla, Narathiwat (expected to be cultivated in the peninsular region) (Fig. 4C). It is cultivated throughout all floristic regions of Thailand.

##### Habitat and ecology.

This species occurs in lowland and swampy habitats, along riverbanks and streams, and in freshwater swamp forests, at elevations from near sea level up to 200 m a.m.s.l.

##### Phenology.

Flowering and fruiting more than once, nearly throughout the year.

##### Conservation status.

*Garcinia schomburgkiana* is distributed from Indo-China to Thailand. It occurs in many localities and has a large EOO of 869,739.19 km^2^ and an AOO of 104 km^2^. In Thailand, this species is naturally distributed in the northern, north-eastern, eastern, and central regions, with an EOO 693,237.84 km^2^ and an AOO of 92 km^2^. Therefore, we assess its conservation status here as LC, consistent with de Kok (2024b).

##### Etymology.

The specific epithet of *Garcinia schomburgkiana* honors Robert Hermann Schomburgk (1804–1865), a German botanist and explorer, and the elder brother of Richard Schomburgk (1811–1891). He was active in Bangkok, Thailand from 1857 to 1864 (Stafleu and Cowan 1985).

##### Vernacular names.

Ka dan (กะดัน) (Nakhon Phanom, from the specimen *A. F. G. Kerr 8421*); Ba dan (บะดัน) (Chiang Mai, from the specimen *T. Bjørnland & T. Schumacher 366*); **Ma dan** (มะดัน) (Bangkok, from the specimen *A. F. G. Kerr s.n*., *A. F. G. Kerr 4335*, *A. F. G. Kerr 4335A*); Ma dan pa (มะดันป่า) (Maha Sarakham, from the specimen *T. Smitinand 10441*).

##### Uses.

*Garcinia schomburgkiana* is cultivated for its fruits and leaves, both of which have a sour taste. The raw fruits, known for their strong sour flavor, are often eaten as a side dish with chili paste or dipped in salt. They are also used as a souring agent in dishes, substituting for lime or tamarind in boiled fish, pork, or beef with *ma dan*, spicy *ma dan* salad, and chili paste. Young leaves are used in red curry with pork and *ma dan* leaves, as well as in smoky, spicy soup with *ma dan* leaves and fried fish. The fruits can also be pickled or preserved as candied fruit. The plant is also commonly grown as an ornamental (from the author’s observations).

In Huai Thap Than District, Si Sa Ket Province, the durable wood of the *ma dan* tree is traditionally used to craft grilling clamps for preparing Thai-style charcoal-grilled chicken (from the author’s observations).

##### Lectotypification.

In the original publication of *Garcinia schomburgkiana* by Pierre (1883), two gatherings were cited: *Schomburgk 1151*, described as a common species in the Chao Phraya River (“Espèce commune dans le delta du Menam”), and *Teysmann* (*Herb. L. Pierre 4150*), noted as a species cultivated in the Bogor Botanical Gardens (“Elle est cultivèe dans le Jardin botanique de Buitenzorg”). Pierre did not indicate a type, nor did he specify the herbaria in which the specimens were housed. Following Art. 9.6 of the ICN (Turland et al. 2018), these specimens are considered syntypes. We located the specimens *Schomburgk s.n*., collected from Thailand (“Siam”) in 1859 at K [K000677697, K000677698] and P [P04701350], and the *Teysmann* specimen (*Herb. L. Pierre 4150*) at P [P04701349]. However, upon examination, the *Schomburgk* specimen appear to bear the number *s.n*. (sine numero), suggesting that the citation of “*Schomburgk 1151*” in the original description may be a transcription error. Jean Baptiste Louis Pierre (1833–1905), a French botanist and director of the Saigon Botanical Garden from 1865 to1877, conducted extensive botanical exploration in Cambodia, Cochinchina, and southern Thailand. Many Pierre’s plant collections were sent to Paris during his lifetime (Stafleu and Cowan 1983). Therefore, the *Schomburgk s.n*. specimen at P [P04701350] is designated here as the lectotype of *G. schomburgkiana*. Although the *Teysmann* specimen (*Herb. L. Pierre 4150*) at P is more complete, the *Schomburgk* specimen was selected for both historical and nomenclatural reasons. The species was named in honor of Schomburgk, and this specimen is directly associated with the collector cited in the protologue. Isolectotypes are designated at K [K000677697, K000677698], following Arts. 9.3 and 9.12 of the ICN (Turland et al. 2018).

##### Notes.

*Garcinia schomburgkiana* is recognized by its habit as a small evergreen tree, monoecious or dioecious, 3–7 m tall, much-branched, with spreading branches; leaves elliptic, narrowly elliptic, or oblong-elliptic, 5–14 × 2.5–4 cm; petals variable in color, pale yellow, yellow, pinkish yellow, yellowish pink, pink, pinkish red, or red; fruits variable in shape, ovoid, broadly ovoid, ellipsoid, broadly ellipsoid, sometimes obovoid, broadly obovoid, or subglobose, 4–9 × 2.5–5 cm, unlobed; and seeds crescent-shaped or semi-ellipsoid, with white fleshy pulp.

##### Additional specimens examined.

**Thailand. Northern**: • Chiang Mai [Ban Pong Noi, Mueang Chiang Mai District, fl., 21 Sep 1978 (as *Garcinia cf. delpyana*), *T. Bjørnland & T. Schumacher 366* (C); • Ban Nam Lat, Chom Thong District, fl., 20 Aug 1980 (as *G. cowa*), *S. Saewa 300* (CMUB); • Locality unspecified, fl. & fr., 5 Sep 1915, cultivated, *Winit 329* (BKF, K [K003964629])]; • Lampang [Thung Kwian, fl., 12 Jul 1995 (as *Garcinia* sp.), *BGO Staff 3942* (QBG)]; **North-Eastern**: • Bueng Kan [Kut Thing Marshland, Non Sombun Subdistrict, Mueang Bueng Kan District (*Assoc. Prof. Dr Sawai Mattapha* personal observation with photos)]; • Nakhon Phanom [That Phanom District, fl., 10 Feb 1924 (as *Garcinia* sp.), *A. F. G. Kerr 8421* (BK, BM, C, K [K003964623], P [P05062003]); • Don Toei Subdistrict, Si Songkhram District, fl., 28 Mar 2010, *P. Karaket, U. Kawatkul & R. Meeboonya 67* (BKF, L [L3810842])]; • Maha Sarakham [Kosum Phisai District, fl., May 1968 (as *Garcinia* sp., *G. fusca*), *T. Smitinand 10441* (BKF, C, K, P [P05062025]); • Ban Muang Yai, Kosum Phisai District, fr., 1 May 2001, *M. Norsangsri, W. Boonchai & W. Nanakorn 1411* (QBG)]; **Eastern**: • Roi Et [Ban Thung Luang, Suwannaphum District, fl., 7 Jun 1982, *Y. Paisooksantivatana & S. Sutheesorn y877-82* (BK); • Si Sa Ket (*C. Ngernsaengsaruay* personal observation); Ubon Ratchathani (*C. Ngernsaengsaruay* personal observation with photos)]; **Central**: • Ang Thong [Wat Klang, Hua Phai Subdistrict, Mueang District, fl., 15 Aug 1971 (as *G. cf. forbesii*), *J. F. Maxwell 71-483* (AAU, BK)]; • Nakhon Pathom [Ban Songkanong, Sam Phran District, fr., 11 Sep 1968, cultivated, *S. Pinnin 292* (BKF)]; • Pathum Thani [Locality unspecified, fl., 30 Jul 1973, *G. Murata & N. Fukuoka T-17323* (L [L2409509]); • Bangkok [Temple, fl., 11 Jul 1920, *A. Marcan 296a* (BM, K [K003964627]); • Temple, fl., 11 Jul 1920, *A. Marcan 296b* (BM, K [K003964626]); • Temple, fl., 30 Jul 1922, cultivated, *A. Marcan 961* (BM, SING); • Khlong San District, fl., 24 Jun 1970, *J. F. Maxwell 70-46* (BK); • Don Mueang Market, fl., 21 Feb 1972, cultivated, *J. F. Maxwell 72-35* (BK, L [L2417730]); • Bang Chak, Bangkok Noi District, fr., 20 Sep 1976, *T. Smitinand s.n*. (BKF); • Weekend market, sterile, *E. Tang 1140* (SING [SING0016760]); • Bang Kruai District, ♂ fl., 5 Aug 2007, *P. Klomsakul 3, 4* (BKF); • Locality unspecified, fl., 4 Jul 1920 (as *Garcinia* sp.), *A. F. G. Kerr s.n*. (BKF); • fl., 11 Jul 1920, *A. F. G. Kerr 4335* (BM, E [E00839794], K [K003964625]); • fr., 11 Jul 1920, *A. F. G. Kerr 4335A* (BM, K [K003964624], P [P04701351]); • fr., 3 Oct 1920, *A. F. G. Kerr s.n*. (BM); • fl., 4 Jul 1921, *A. F. G. Kerr 28* (BM); • sterile, 23 Jul 1979, *L. Muangnoicharoen s.n*. (BKF)]; **Peninsular**: • Surat Thani [Khao Nam Chong, Tha Chana District, fl., 10 Jul 1966, *Sakol 1272* (BK)]; • Songkhla [Ban Hu Rae, Hat Yai District, ♂ fl., 26 Jun 1986 (as *G. parvifolia*), *G. Junchote 1* (AAU, PSU); • Prince of Songkhla University, Hat Yai Campus, ♂ fl., 27 Sep 1985 (as *G. cowa*), cultivated, *J. F. Maxwell 85-910* (PSU); • ibid., ♂ fl., 5 Jan 1989, cultivated, *Sathon 3* (C); • ibid., ♂ fl., 17 Dec 2003, cultivated, *A. Sloth 585* (AAU, BKF)]; • Narathiwat [To Daeng Peat Swamp Forest, sterile, Apr 1998, *K. Chayamarit 1507* (BKF)]; • Region and Province unspecified [Locality unspecified, sterile, 11 Oct 1926, *M. C. Lakshnakara 283* (K [K003964628])]; • From Thailand, cultivated in Hort. Bogor, Java, Indonesia, 1903, *Unknown s.n*. (US [US02961239]).

## Supplementary Material

XML Treatment for Garcinia cowa

XML Treatment for Garcinia cowa

XML Treatment for Garcinia oliveri

XML Treatment for Garcinia schomburgkiana

## Additional information

### Use of AI

No use of AI was reported.

### Author ORCIDs

Chatchai Ngernsaengsaruay https://orcid.org/0000-0002-7131-976X

Pichet Chanton https://orcid.org/0009-0001-7325-6109

### Data availability

All of the data that support the findings of this study are available in the main text.
